# KIAA1429 contributes to liver cancer progression through N6-methyladenosine-dependent post-transcriptional modification of GATA3

**DOI:** 10.1186/s12943-019-1106-z

**Published:** 2019-12-19

**Authors:** Tian Lan, Hui Li, Delin Zhang, Lin Xu, Hailing Liu, Xiangyong Hao, Xiaokai Yan, Haotian Liao, Xiangzheng Chen, Kunlin Xie, Jiaxin Li, Mingheng Liao, Jiwei Huang, Kefei Yuan, Yong Zeng, Hong Wu

**Affiliations:** 1 Department of Liver Surgery & Liver Transplantation, State Key Laboratory of Biotherapy and Cancer Center, West China Hospital, Sichuan University and Collaborative Innovation Center of Biotherapy, Chengdu, 610041 China; 2 Department of Hepatobiliary Surgery, Guizhou Provincial People’s Hospital, Guiyang, 550002 China; 3 Laboratory of Liver Surgery, State Key Laboratory of Biotherapy and Cancer Center, West China Hospital, Sichuan University and Collaborative Innovation Center of Biotherapy, Chengdu, 610041 China; 4 Department of General Surgery, Gansu Provincial Hospital, Lanzhou, 730000 China

**Keywords:** Hepatocellular carcinoma, N6-methyladenosine, KIAA1429, GATA3, GATA3-AS

## Abstract

**Background:**

N6-methyladenosine (m6A) modification, the most abundant internal methylation of eukaryotic RNA transcripts, is critically implicated in RNA processing. As the largest known component in the m6A methyltransferase complex, KIAA1429 plays a vital role in m6A methylation. However, its function and mechanism in hepatocellular carcinoma (HCC) remain poorly defined.

**Methods:**

Quantitative PCR, western blot and immunohistochemistry were used to measure the expression of KIAA1429 in HCC. The effects of KIAA1429 on the malignant phenotypes of hepatoma cells were examined in vitro *and* in vivo. MeRIP-seq, RIP-seq and RNA-seq were performed to identify the target genes of KIAA1429.

**Results:**

KIAA1429 was considerably upregulated in HCC tissues. High expression of KIAA1429 was associated with poor prognosis among HCC patients. Silencing KIAA1429 suppressed cell proliferation and metastasis in vitro and in vivo. GATA3 was identified as the direct downstream target of KIAA1429-mediated m6A modification. KIAA1429 induced m6A methylation on the 3′ UTR of GATA3 pre-mRNA, leading to the separation of the RNA-binding protein HuR and the degradation of GATA3 pre-mRNA. Strikingly, a long noncoding RNA (lncRNA) GATA3-AS, transcribed from the antisense strand of the GATA3 gene, functioned as a *cis*-acting element for the preferential interaction of KIAA1429 with GATA3 pre-mRNA. Accordingly, we found that the tumor growth and metastasis driven by KIAA1429 or GATA3-AS were mediated by GATA3.

**Conclusion:**

Our study proposed a complex KIAA1429-GATA3 regulatory model based on m6A modification and provided insights into the epi-transcriptomic dysregulation in hepatocarcinogenesis and metastasis.

## Background

Hepatocellular carcinoma (HCC), the most common subtype of primary liver malignancy, serves as the third leading cause of cancer-related mortality globally [1]. A high postsurgical recurrence rate and metastasis lead to the poor prognosis of HCC patients. Unfortunately, limited effective treatments are available and patients often miss the optimal opportunities for clinical interventions, because HCC is frequently diagnosed at the advanced stages [2]. As the molecular mechanisms underlying HCC pathogenesis have not yet been completely understood, elucidating the pivotal cancer-promoting events would contribute to the comprehension of hepatocarcinogenesis and the development of novel effective targeted treatments.

N6-methyladenosine (m6A) modification is the most abundant internal methylation of RNA transcripts in eukaryotes [3]. In mammalian cells, m6A modification is a reversible process regulated by m6A WERs (writers, erasers and readers). The formation of m6A methylation is catalyzed by the methyltransferase complex that consist of m6A writers, among which METTL3 (methyltransferase-like 3), METTL14 (methyltransferase-like 14), WTAP (WT1-associated protein) and KIAA1429 (VIRMA, vir-Like m6A methyltransferase associated) play key roles [4]. Conversely, ALKBH5 (a-ketoglutarate-dependent dioxygenase alkB homolog 5) and FTO (fat-mass and obesity-associated protein) act as erasers to reverse the m6A methylation [5]. Several m6A-binding proteins with the YTH domain, containing YTHDF1, YTHDF2, YTHDF3 and YTHDC1, function as the readers to modulate translation and mediate degradation of m6A-modified RNAs [6]. Mounting evidence has demonstrated that m6A modification exerts significant and comprehensive effects on diverse biological regulatory processes, including transcription splicing, RNA stability, translation efficiency, embryonic stem cell maintenance and specification, cell fate determination, T-cell homeostasis, neuronal functions and sex determination [7–9]. Recently, numerous studies have described the crucial role of m6A machinery in different human cancers. For instance, ALKBH5 has been shown to sustain the tumorigenicity of glioblastoma stem-like cells by demethylating FOXM1 nascent transcripts and thus enhancing FOXM1 expression [10]. METTL3 has been identified as an oncogenic driver in HCC, which promotes HCC tumorigenicity and metastasis by repressing SOCS expression in an m6A-YTHDF2-dependent manner [11]. Notably, it is reported that KIAA1429 knockdown results in a median ∼4-fold decrease in m6A peak scores, which is substantially and significantly more prominent than that achieved upon knockdown of either METTL3 or METTL14 [12], suggesting the importance of KIAA1429 in the methyltransferase complex. However, the precise functions of KIAA1429 and the underlying regulatory mechanisms in HCC remain poorly understood.

This study unveiled the potential role of KIAA1429 in the development and progression of HCC. KIAA1429 was significantly upregulated in HCC tissues and associated with the prognosis of HCC patients. Through the performance of high-throughput methylated RNA immunoprecipitation sequencing (MeRIP-seq), RNA immunoprecipitation sequencing (RIP-seq) and RNA-seq, KIAA1429 was found to mediate the m6A methylation of GATA3 pre-mRNA, leading to altered GATA3 expression and thereby facilitating the malignant phenotypes of hepatoma cells. Our observations explored an epi-transcriptomic cause of HCC pathogenesis and characterized KIAA1429 as a promising biomarker with diagnostic and therapeutic significance.

## Methods

### Human samples and cell lines

HCC tissues and adjacent normal tissues were acquired from 70 HCC patients undergoing curative surgical resections in the West China Hospital (Sichuan University, Chengdu, China). The protocols used in this study were approved by the Ethical Review Committees of Sichuan University, and written informed consent was provided from all patients. Huh-7, Hep3B, HepG2, SK-Hep1, HCCLM3 and SNU-182 cell lines were purchased from the Shanghai Cell Bank Type Culture Collection Committee (CBTCCC, Shanghai, China). SNU-449 and HEK293T cell lines were obtained from the American Type Culture Collection (ATCC, Manassas, Virginia, USA). All cell lines were characterized by short tandem repeat (STR) analysis by third-party biology services (Feiouer Biology Co., Ltd., Chengdu, China).

### RNA-sequencing (RNA-seq)

RNA degradation and contamination were monitored on 1% agarose gels. RNA purity was checked using a NanoPhotometer® spectrophotometer (IMPLEN, CA, USA). RNA concentration was measured using the Qubit®RNA Assay Kit in a Qubit® 2.0 Fluorometer (Life Technologies, CA, USA). RNA integrity was assessed using the RNA Nano 6000 Assay Kit of the Bioanalyzer 2100 system (Agilent Technologies, CA, USA). Sequencing libraries were generated using the NEBNext® Ultra™ RNA Library Prep Kit for Illumina® (NEB, USA) following the manufacturer’s recommendations. First strand cDNA was synthesized using random hexamer primer and M-MuLV reverse transcriptase (RNase H-). Second-strand cDNA synthesis was subsequently performed using DNA polymerase I and RNase H. The library quality was assessed on the Agilent Bioanalyzer 2100 system. Clustering of the index-coded samples was performed on a cBot Cluster Generation System using TruSeq PE Cluster Kit v3-cBot-HS (Illumina) according to the manufacturer’s instructions. After cluster generation, the library preparations were sequenced on an Illumina Hiseq 2500 platform and 125 bp paired-end reads were generated. Reference genome and gene model annotation files were downloaded from the genome website browser (NCBI/UCSC/Ensembl) directly. Indexes of the reference genome were built using Bowtie v2.0.6 [13], and paired-end clean reads were aligned to the reference genome using TopHat v2.0.9 [14]. HTSeq v0.6.1 was used to count the read numbers mapped to each gene. Differential expression analysis between two groups was performed using the DESeq R package based on the negative binomial distribution. The resulting *P* values were adjusted using Benjamini and Hochberg’s approach for controlling the false discovery rate (FDR). Genes with |log_2_[fold change (FC)]| > 1 and FDR < 0.05 found by DESeq were considered to be differentially expressed [15].

### RNA Immunoprecipitation sequencing (RIP-seq)

RIP was performed using the Magna RIP™ RNA-binding Protein Immunoprecipitation Kit (Millipore, Massachusetts, USA), according to the manufacturer’s protocol. Briefly, magnetic beads coated with 10 μg of specific antibodies against KIAA1429 (Cell Signaling Technology, Boston, USA) or normal IgG (Millipore, Massachusetts, USA) were incubated with prepared indicated cell lysates overnight at 4 °C. Washed RNA-protein complexes were treated with proteinase K digestion buffer. The coprecipitated RNAs were purified with phenol: chloroform: isoamyl alcohol and subsequently subjected to purity and concentration analysis using NanoDrop ND-1000. Intact mRNA was isolated from total RNA samples using an Arraystar Seq-Star™ poly (A) mRNA Isolation Kit (Arraystar, MD, USA), followed by chemical mRNA fragmentation. First-strand cDNA was synthesized using random hexamer primer and second-strand cDNA synthesis was subsequently performed using DNA polymerase I before adaptor ligation and PCR amplification. The library quality was assessed on an Agilent Bioanalyzer 2100 system. Clustered libraries were loaded onto a reagent cartridge and forwarded for sequencing runs on an Illumina Hiseq 4000 system by Aksomics (Shanghai, China). Sequencing reads were aligned to the human genome GRCh37/hg19 by HISAT2 [16]. The resulting *P* values were adjusted using Benjamini and Hochberg’s approach for controlling the false discovery rate (FDR). Genes with Log_2_[fold change (FC)] > 1 and FDR < 0.05 found by DESeq were considered to be significant enrichment [15, 17].

### Methylated RNA immunoprecipitation sequencing (MeRIP-seq)

MeRIP and library preparation were conducted according to a previously published protocol with minor revisions [18]. One hundred twenty micrograms of purified total RNA was evaluated with a NanoDrop ND-1000. Intact mRNA was isolated from total RNA samples using an Arraystar Seq-Star™ poly (A) mRNA Isolation Kit (Arrarystar, MD, USA) following the manufacturer’s instructions and was chemically fragmented into 100-nucleotide-long fragments by incubation in fragmentation buffer. Fragmented mRNA was immunoprecipitated with anti-N6-methyladenosine (m6A) antibody (Synaptic Systems, Goettingen, Germany), and 1/10 of the fragmented mRNA was kept as input. RNA-seq libraries were prepared using a KAPA Stranded mRNA-seq Kit (Illumina, CA, USA). The completed libraries, qualified by an Agilent 2100 Bioanalyzer, were denatured and diluted to the loading volume of a loading concentration. Clustered libraries were loaded onto a reagent cartridge and forwarded for sequencing runs on an Illumina Hiseq 4000 system by Aksomics (Shanghai, China). Sequencing peaks were annotated to the Ensembl database. Sequence motifs were identified using MEME-ChIP analysis [19].

### Statistical analysis

Data were presented as the mean ± standard error of the mean (SEM). All results were representative of three separate experiments. All statistical analyses were performed using GraphPad Prism 8 software (GraphPad Software) and SPSS version 25.0 software (SPSS, Inc., Chicago, IL). The median KIAA1429 or GATA3 expression was used as a cut-off value for grouping. The survival curves were measured by the Kaplan-Meier method, and the differences were evaluated by the log-rank test. Univariate and multivariate Cox proportional hazards regression models were utilized to assess independent factors. Statistical significance was indicated by *P* values less than 0.05. *P < 0.05, **P < 0.01, ***P < 0.001.

Additional materials and methods are described in Additional file 1. Additional file legends are described in Additional file 17.

## Results

### KIAA1429 is elevated in HCC tissues and associated with poor prognosis

**Fig. 1:**
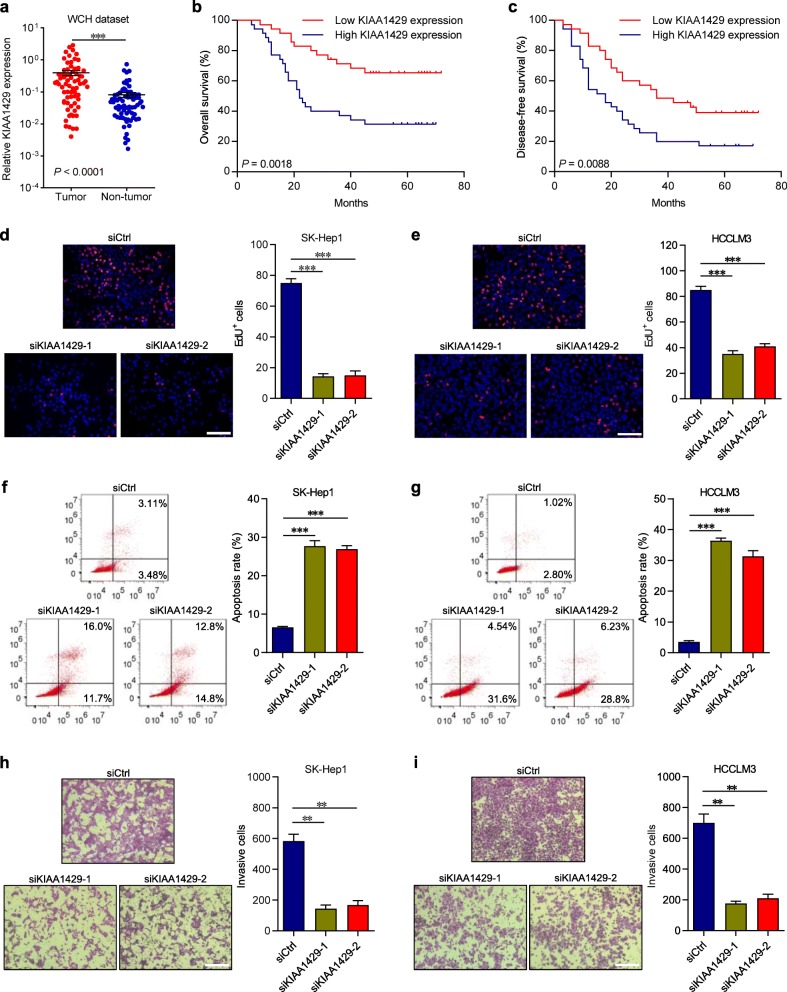
KIAA1429 predicts poor prognosis of HCC patients and is required for cell proliferation and invasion. **a** KIAA1429 expression in 70 pairs of HCC tissues and adjacent normal tissues from WCH dataset by using qPCR. **b-c** Kaplan-Meier analyses of the correlations between KIAA1429 expression and overall survival or disease-free survival of 70 HCC patients. The median expression level was used as the cutoff. Values are expressed as the median with interquartile range. **d-e,** EdU immunofluorescence staining assays for SK-Hep1 and HCCLM3 cells transfected with KIAA1429 siRNAs or the control. Scale bars = 100 μm. **f** and **g,** Cell apoptosis was measured by FITC-Annexin V and PI staining in SK-Hep1 and HCCLM3 cells transfected with KIAA1429 siRNAs or the control, followed by flow cytometric analysis. **h** and **i,** Transwell invasion assays for SK-Hep1 and HCCLM3 cells transfected with KIAA1429 siRNAs or the control. Scale bars = 100 μm. Data are presented as mean ± SEM. **P < 0.01, ***P < 0.001

To investigate the expression level of KIAA1429 in HCC, we first queried The Cancer Genome Atlas (TCGA) data repository of 50 paired HCC samples [20]. The results showed that KIAA1429 was significantly higher in HCC tissues than in adjacent normal tissues, and HCC patients with elevated expression of KIAA1429 had poor overall survival and disease-free survival (Additional file 2: Figures S1a-c). Subsequently, we confirmed KIAA1429 upregulation in 70 pairs of HCC tissues from the West China Hospital (WCH) dataset by quantitative PCR (qPCR) analysis, consistently demonstrating that the expression of KIAA1429 was dramatically upregulated in HCC tissues compared with that in adjacent normal tissues (Fig. 1a). Importantly, the areas under the receiver operating characteristic curves (ROCs) for KIAA1429 expression in the TCGA dataset and WCH dataset were 85 and 75%, respectively (Additional file 2: Figures S1d-e), suggesting a relatively high diagnostic value of KIAA1429 in HCC. Additionally, immunohistochemical (IHC) staining and western blot analysis further validated the upregulation of KIAA1429 in HCC, which was consistent with the observations at the mRNA level (Additional file 2: Figures S1f-g). Afterwards, the correlations between KIAA1429 expression and clinical characteristics were analyzed in 70 HCC tissues, revealing that high expression of KIAA1429 was significantly associated with tumor size (*P* = 0.0303), serum AFP (*P* = 0.0157), microvascular invasion (*P* = 0.0168), TNM stage (*P* = 0.0220) and BCLC stage (*P* = 0.0051) (Additional file 3: Table S1). Kaplan-Meier analysis illustrated that increased KIAA1429 expression was statistically associated with shorter overall survival and disease-free survival (Figs. 1b, c). Collectively, these results suggested that KIAA1429 might be implicated in HCC progression and could be a potential prognostic indicator for patients with HCC.

### KIAA1429 enhances tumor growth and metastasis in vitro and in vivo

Next, the expression of KIAA1429 was examined in seven human hepatoma cell lines, among which SK-Hep1 and HCCLM3 showed relatively high KIAA1429 expression, whereas Huh-7 and SNU-182 displayed considerably lower KIAA1429 expression (Additional file 2: Figure S1 h).

To explore the oncogenic role of KIAA1429 in cell growth and metastasis, we evaluated the effects of KIAA1429 on cell phenotypes. Knockdown of KIAA1429 (Additional file 4: Figures S2a-c) significantly suppressed SK-Hep1 and HCCLM3 proliferation, cell cycle and apoptosis resistance (Figs. 1d-g and Additional file 4: Figures S2d-g). Furthermore, silencing KIAA1429 markedly impaired the invasive and migratory capacities of SK-Hep1 and HCCLM3 cells (Figs. 1h-i and Additional file 4: Figures S2 h-i).

**Fig. 2:**
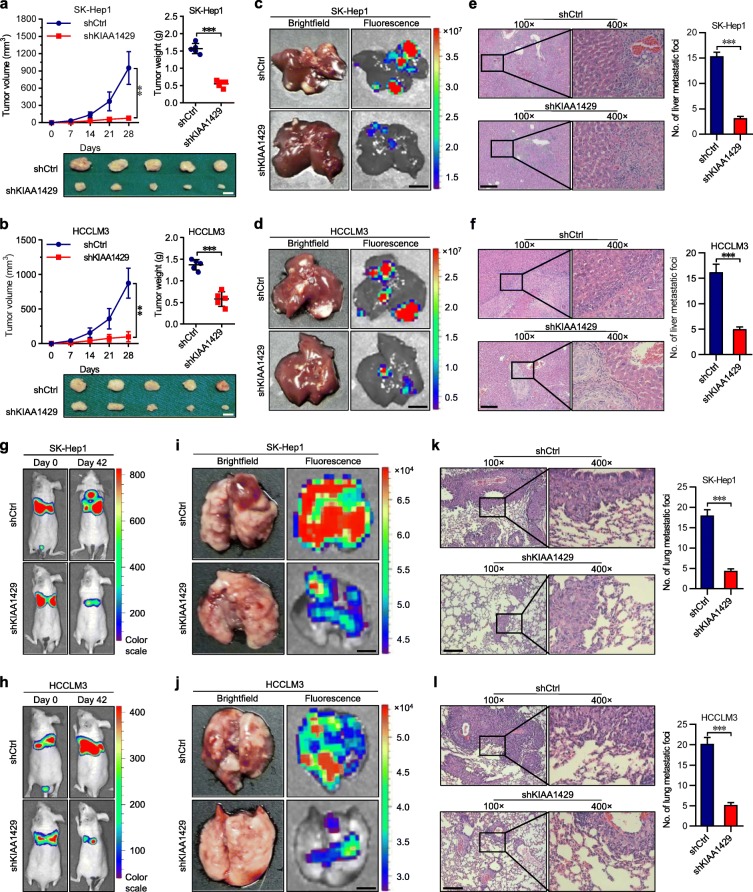
knockdown of KIAA1429 impairs the tumor growth and metastasis in vivo. **a** and **b** Tumor volume and tumor weight in subcutaneous xenografts models with indicated SK-Hep1 and HCCLM3 cells. Scale bars = 5 mm. **c** and **d** Representative brightfield and fluorescence photographs of intrahepatic metastatic nodules in orthotopic implantation models with indicated SK-Hep1 and HCCLM3 cells. Scale bars = 5 mm. **e** and **f** Representative microscopic views of intrahepatic metastatic foci from indicated SK-Hep1 and HCCLM3 cells in tissue sections of livers using HE staining. Scale bars = 100 μm. **g** and **h** Representative bioluminescence photographs of the murine tail vein injection lung metastasis models at indicated times by imaging with the IVIS@ Lumina II system. **i** and **j** Representative brightfield and fluorescence photographs of pulmonary metastatic nodules in lung metastasis models with indicated SK-Hep1 and HCCLM3 cells. Scale bars = 2 mm. **k** and **l** Representative microscopic views of pulmonary metastatic foci from indicated SK-Hep1 and HCCLM3 cells in tissue sections of lungs using HE staining. Scale bars = 100 μm. Data are presented as mean ± SEM. **P < 0.01, ***P < 0.001

To further investigate the tumorigenic function of KIAA1429 in vivo, SK-Hep1 and HCCLM3 cells infected with LV-shKIAA1429 or LV-shCtrl were subcutaneously inoculated into nude mice. Both the tumor volume and weight showed remarkable reduction in the LV-shKIAA1429 group compared with the LV-shCtrl group (Figs. 2a-b), indicating that stable depletion of KIAA1429 effectively inhibited tumor growth in vivo. Then, we assessed the impacts of KIAA1429 on tumor metastasis in vivo. Indicated cells infected with LV-shKIAA1429 or LV-shCtrl were transplanted into the livers of nude mice to establish liver orthotopic-implanted models. Six weeks after transplantation, the fluorescence signal intensities of liver metastatic nodules in the LV-shKIAA1429 group were significantly lower than those in the LV-shCtrl group (Figs. 2c-d and Additional file 5: Figures S3a-b). Hematoxylin and eosin (HE) staining indicated that the number of metastatic foci in the LV-shKIAA1429 group substantially declined in tissue sections of the livers (Figs. 2e-f), implying that KIAA1429 strengthened the intrahepatic metastatic ability of hepatoma cells. Moreover, indicated cells labeled with firefly luciferase were injected into the tail vein of nude mice to construct lung metastasis models. Compared with the LV-shCtrl group, the LV-shKIAA1429 group was associated with considerably decreased bioluminescence signal intensities in mice (Figs. 2g-h), dramatically reduced fluorescence signal intensities of lung metastatic nodules (Figs. 2i-j and Additional file 5: Figures S3c-d) and fewer metastatic foci in tissue sections of the lungs (Figs. 2k-l), illustrating that the lung metastatic potential of hepatoma cells could be facilitated by KIAA1429.

In addition, we further ascertained the effects of KIAA1429 upregulation on the malignant behaviors of hepatoma cells. Overexpression of KIAA1429 significantly boosted the cell cycle, proliferation and apoptotic resistance of Huh-7 and SNU-182 cells (Additional file 5: Figures S3e-l). Additionally, upregulation of KIAA1429 statistically increased the invasiveness and migration of Huh-7 and SNU-182 cells (Additional file 5: Figures S3 m-n and Additional file 6: Figures S4a-b). Taken together, these findings indicated that KIAA1429 functions as an oncogenic driver in cell proliferation and metastasis.

### Multidimensional sequencing identifies GATA3 as a downstream target of KIAA1429

**Fig. 3:**
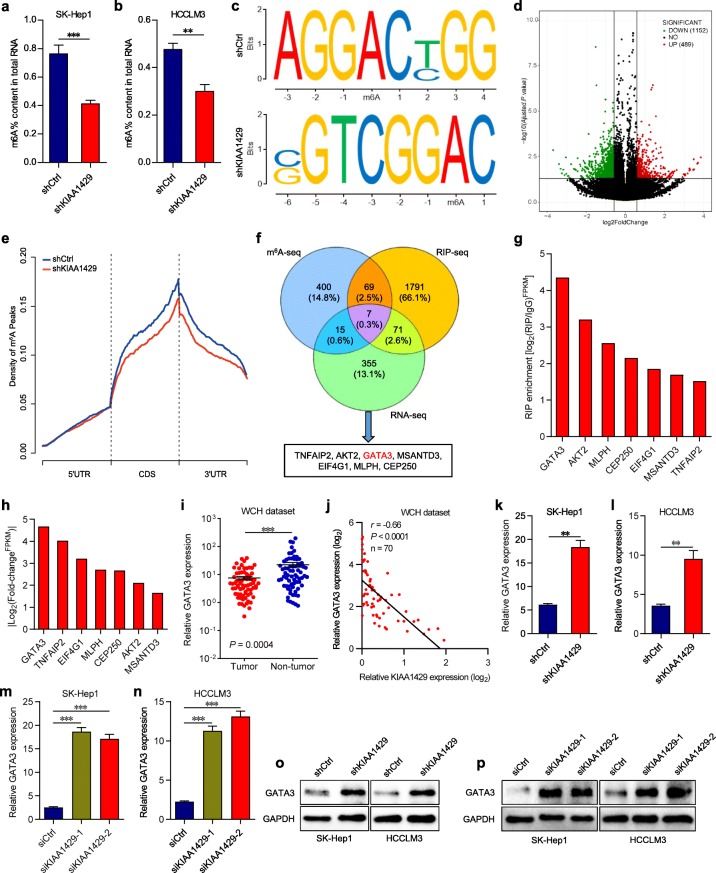
Characterization of m6A modification and identification of KIAA1429 downstream target. **a** and **b** Relative m6A levels on poly-A selected RNA in indicated SK-Hep1 and HCCLM3 cells were assessed by the EpiQuik m6A RNA Methylation Quantification Kit (Colorimetric). **c** Top sequence motif identified from MeRIP-seq peaks in control and KIAA1429-depleted cells. **d** Volcano plot of altered m6A peaks identified in MeRIP-seq in control and KIAA1429-depleted cells. **e** Distribution of reduced m6A peaks generated by KIAA1429 inhibition across all mRNAs. **f** Schematic of the selection for the direct downstream target of KIAA1429. **g** RIP-seq of the enrichment of candidate mRNA transcripts on KIAA1429 relative to IgG. **h** RNA-seq of the fold change of candidate mRNA transcripts caused by KIAA1429 depletion. **i** GATA3 expression in 70 pairs of HCC tissues and adjacent normal tissues from WCH dataset by using qPCR. **j** Scatter plots of KIAA1429 versus GATA3 expression in WCH data repository. Pearson correlation coefficients (r) and *P* values are shown. **k-l** GATA3 expression in SK-Hep1 and HCCLM3 cells with stable KIAA1429 knockdown by using qPCR. **m** and **n** GATA3 expression in SK-Hep1 and HCCLM3 cells with transient KIAA1429 knockdown by using qPCR. **o** and **p** Western blot analysis of GATA3 expression in SK-Hep1 and HCCLM3 cells with stable and transient KIAA1429 knockdown. Data are presented as mean ± SEM. **P < 0.01, ***P < 0.001

Given that KIAA1429 is one of the m6A writers, we tested whether KIAA1429 exerted regulatory effects on the global m6A modification in hepatoma cells. As expected, significantly lower global m6A levels were observed in KIAA1429-inhibited SK-Hep1 and HCCLM3 cells (Figs. 3a-b), validating the m6A methylation activity of KIAA1429 in hepatoma cells. Afterwards, to gain insights into the regulatory implications of KIAA1429 in terms of comprehensive gene expression, RNA-seq was employed to compare the expression profiles between control and KIAA1429 stable knockdown cells. A total of 448 genes were differentially expressed by at least 2-fold in stable KIAA1429-inhibited cells, which were found to be significantly enriched in gene sets involved in most RNA-specific processes (RNA catabolic process, RNA metabolic process, RNA transport, RNA localization and translation), cell cycle, cell death, apoptosis and intracellular signaling pathways by Gene Ontology (GO) analysis (Additional file 6: Figure S4c). Notably, Kyoto Encyclopedia of Genes and Genomes (KEGG) analysis indicated that Toll-like receptor, chemokine and various cancer-associated signaling pathways were affected by KIAA1429 downregulation (Additional file 6: Figure S4d), confirming the role of KIAA1429 in promoting liver cancer progression.

To elucidate whether the altered gene expression was ascribed to KIAA1429-mediated m6A methylation, MeRIP-seq was performed to compare the global profiling of m6A target genes between control and stable KIAA1429-inhibited cells. The “GGAC” sequence motif was verified to be highly enriched in m6A-immunoprecipitated RNAs (Fig. 3c), which was consistent with previously reported studies [9, 10]. In total, 1152 and 489 m6A peaks presented statistical decrease and increase, respectively, in KIAA1429 knockdown cells relative to control cells (Fig. 3d). Since KIAA1429 positively mediates m6A modification, the 1152 decreased peaks are theoretically anticipated to include genuine targets of KIAA1429. Therefore, we focused on the mRNA transcripts with these reduced m6A peaks that were found to be predominantly localized in the 3′ untranslated region (3′ UTR) and near the stop codon by the metagene analysis (Fig. 3e) [10]. Next, the association between these peaks and differentially expressed genes identified by RNA-seq was analyzed. Filtering the 1152 decreased m6A peaks within the 448 genes with 2-fold change led to the identification of 22 candidate genes (Fig. 3f), suggesting that knockdown KIAA1429 might reduce the m6a levels of these 22 gene transcripts and thus result in the altered expressions of these transcripts. To further confirm the transcripts directly regulated by KIAA1429, RIP-seq experiment using endogenous anti-KIAA1429 antibody was implemented to ascertain the transcripts bound by KIAA1429, identifying 2171 transcripts with 2-fold enrichment in the KIAA1429-immunoprecipitated group. At the intersection of RNA-seq, RIP-seq and MeRIP-seq, 7 genes (TNFAIP2, AKT2, GATA3, MSANTD3, EIF4G1, MLPH and CEP250) were screened out (Fig. 3f), suggesting that KIAA1429 might bind to these 7 transcripts and directly modulate their m6a levels, leading to the altered expressions of these transcripts. Among them, GATA binding protein 3 (GATA3) showed the highest enrichment in KIAA1429-immunoprecipitated RNAs, and its expression demonstrated the most significant shift after KIAA1429 knockdown (Figs. 3g-h), suggesting that KIAA429-mediated m6a modification might mainly focus on GATA3 transcript. With the above evidence, we deduced that GATA3 might act as the direct downstream target of KIAA1429-mediated m6A methylation.

GATA3 has been proven to be a powerful tumor suppressor gene [21]. We thereby examined the expression level of GATA3 in 70 pairs of HCC samples from the WCH dataset. Accordingly, GATA3 expression was significantly lower in HCC tissues than in adjacent normal tissues (Fig. 3i) and was considerably correlated with KIAA1429 expression (Fig. 3j), implying the regulatory relationship of GATA3 expression by KIAA1429. Subsequently, we measured the RNA and protein expression of GATA3 upon silencing KIAA1429 by transfection with siRNAs or infection with lentivirus-packaged shRNA. Both transient and stable KIAA1429 knockdown resulted in statistically elevated GATA3 abundance at the RNA and protein levels (Figs. 3k-p), indicating that KIAA1429 is the direct upstream regulator of GATA3.

### GATA3 precursor mRNA (pre-mRNA) serves as the substrate of KIAA1429-mediated m6A modification

To clarify the molecular mechanism underlying the regulatory effects of KIAA1429 on GATA3, we initially examined whether KIAA1429 would accelerate GATA3 mRNA degradation or impair GATA3 gene transcription. Intriguingly, after treatment with actinomycin D, KIAA1429-inhibited cells were not associated with altered GATA3 mRNA stability within 24 h but displayed significantly enhanced GATA3 mRNA stability at 48 h, suggesting that KIAA1429 might indirectly promote GATA3 mRNA degradation (Additional file 7: Figures S5a-b). Additionally, the promoter activity of the GATA3 gene remained unchanged in control and KIAA1429-inhibited cells (Additional file 7: Figures S5c-d). The results raised the possibility that KIAA1429 depletion might lead to the increase of GATA3 pre-mRNA and subsequently a significant difference in the expression of GATA3 mRNA. Indeed, GATA3 pre-mRNA expression experienced significant growth following KIAA1429 siRNA transfection (Additional file 7: Figures S5e-f).

It has been reported that m6A modification is involved in the RNA splicing process [22]. To explore whether KIAA1429 was able to act on GATA3 pre-mRNA, the subcellular localization of these two molecules was detected using subcellular RNA and protein fractionation. The data demonstrated that both GATA3 pre-mRNA and KIAA1429 were predominantly situated in the nucleus (Additional file 7: Figures S5 g-i). Furthermore, RIP assays confirmed the remarkably high enrichment of GATA3 pre-mRNA in KIAA1429 immunoprecipitates, revealing that KIAA1429 co-localized and interacted with GATA3 pre-mRNA (Additional file 7: Figures S5j-k). Then, we employed MeRIP-qPCR to determine the m6A methylation levels of GATA3 pre-mRNA upon KIAA1429 silencing. The m6A level of GATA3 pre-mRNA presented a statistical reduction in KIAA1429 knockdown cells relative to control cells (Additional file 7: Figures S5 l-m). Together, these observations suggested that KIAA1429 mediates the m6A modification of GATA3 pre-mRNA.

### GATA3 3′ UTR is responsible for KIAA1429-mediated m6A methylation

**Fig. 4:**
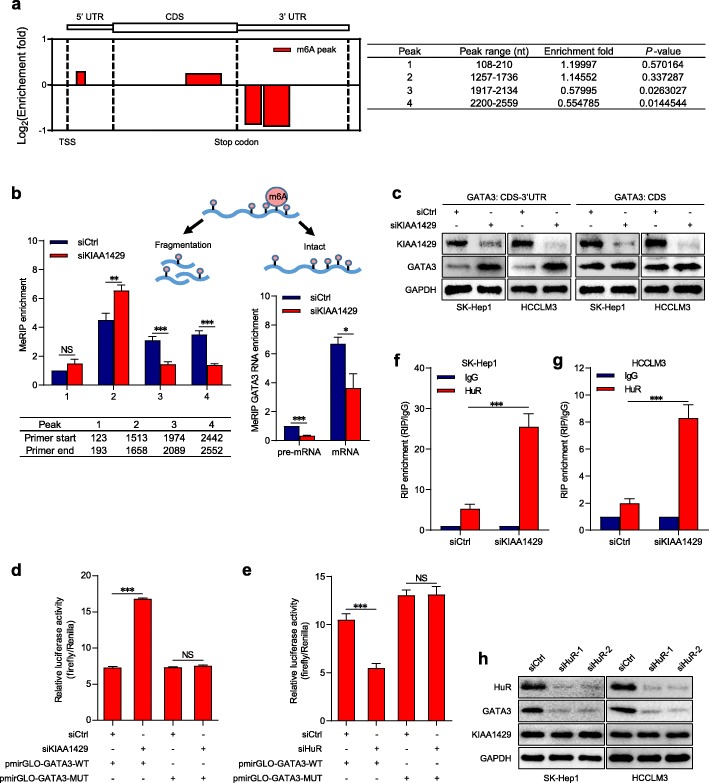
The GATA3 3′ UTR maintains the KIAA1429-mediated m6A regulation. **a** MeRIP-seq of the distribution of m6A peaks along GATA3 mRNA in KIAA1429-inhibited cells. **b** MeRIP-qPCR analysis of fragmented or intact GATA3 mRNA or GATA3 pre-mRNA in control and KIAA1429-depleted cells. **c** Western blot analysis of GATA3 expression in SK-Hep1 and HCCLM3 cells co-transfected with GATA3 CDS-3′ UTR or GATA3 CDS vector and KIAA1429 siRNAs or the control. **d** Relative luciferase activity in HEK293T cells co-transfected with luciferase reporter pmirGLO-GATA3-WT or pmirGLO-GATA3-MUT and KIAA1429 siRNAs or the control. Data are shown as the relative ratio of firefly luciferase activity to renilla luciferase activity. **e** Relative luciferase activity in HEK293T cells co-transfected with luciferase reporter pmirGLO-GATA3-WT or pmirGLO-GATA3-MUT and HuR siRNAs or the control. Data are shown as the relative ratio of firefly luciferase activity to renilla luciferase activity. **f** and **g** RIP-qPCR analysis of the enrichment of GATA3 pre-mRNA on HuR relative to IgG in control and KIAA1429-depleted cells. **h** Western blot analysis of HuR, GATA3 and KIAA1429 expressions in SK-Hep1 and HCCLM3 cells transfected with HuR siRNAs or the control. Data are presented as mean ± SEM. NS: not significant; *P < 0.05, **P < 0.01, ***P < 0.001

Based on the comparative analysis of MeRIP-seq data in control and KIAA1429-inhibited cells, four m6A peaks were found in GATA3 mRNA, including one peak on the 5′ UTR, one peak on the coding sequence (CDS) and two statistically decreased peaks on the 3′ UTR (Fig. 4a). The m6A levels of these peaks were further validated by MeRIP-qPCR with specific primers in fragmented RNA samples (Fig. 4b). Thereafter, we analyzed the intact GATA3 pre-mRNA and GATA3 mRNA by MeRIP-qPCR, which indicated that m6A levels simultaneously showed a significant decline following KIAA1429 depletion (Fig. 4b). Moreover, compared with mature mRNA, GATA3 pre-mRNA was associated with a relatively lower m6A proportion (Fig. 4), demonstrating that m6A-modified GATA3 pre-mRNA was more likely to be degraded. Thus, we inferred that KIAA1429 facilitated the m6A methylation of GATA3 3′ UTR and then the detectable reduction of GATA3 pre-mRNA expression. First, to verify the importance of GATA3 3′ UTR in this regulatory model, expression vectors containing the GATA3 CDS with 3′ UTR (CDS-3′ UTR) or CDS alone were constructed and subsequently cotransfected with siRNAs against KIAA1429 into SK-Hep1 and HCCLM3 cells. The data illustrated that KIAA1429 inhibition resulted in significantly increased expression of GATA3 on the basis of transfection with the GATA3 CDS-3′ UTR vector rather than the GATA3 CDS alone vector (Fig. 4c and Additional file 8: Figures S6a-b), indicating that 3′ UTR was indispensable for the modulatory effects of KIAA1429 on GATA3. Second, we evaluated the effects of m6A incorporation on the GATA3 3′ UTR. The pmirGLO-GATA3-WT luciferase reporter containing GATA3 3′ UTR after the firefly luciferase (Fluc) coding sequence was synthesized and cytosine (C) bases were substituted for the adenosine (A) bases in the m6A motif sequences to establish the pmirGLO-GATA3-MUT luciferase reporter. The luciferase activity of pmirGLO-GATA3-WT showed a marked rise upon KIAA1429 knockdown (Fig. 4d). In contrast, pmirGLO-GATA3-MUT showed no response to KIAA1429 silencing (Fig. 4d), which revealed that the regulation of GATA3 expression was controlled by KIAA1429-mediated m6A modification on the GATA3 3′ UTR.

### KIAA1429 abolishes the binding of HuR to GATA3 pre-mRNA

Because m6A-modified GATA3 pre-mRNA was less stable, we investigated which molecules played crucial roles in the degradation of GATA3 pre-mRNA under the influence of m6A methylation. The RNA-binding protein HuR, which reportedly couples pre-mRNA processing and mRNA stability [23], has been identified to stabilize its bound RNAs without m6A modification [10, 24]. To clarify whether HuR was critically involved in our regulatory model, luciferase reporter assays were first conducted. Upon HuR inhibition (Additional file 8: Figures S6c-d), luciferase activity was significantly decreased in cells transfected with the pmirGLO-GATA3-WT but not the pmirGLO-GATA3-MUT (Fig. 4e), suggesting that HuR disfavored stabilization of m6A-modified GATA3 3′ UTR-fused RNA. Next, RIP assays confirmed the interaction between HuR and GATA3 pre-mRNA, which was substantially consolidated by depleting KIAA1429 (Figs. 4f-g). In contrast, no significantly high enrichment of GATA3 mRNA in HuR immunoprecipitates was observed (Additional file 8: Figures S6e-f). Eventually, we tested the expressions of GATA3, GATA3 pre-mRNA and KIAA1429 in cells transfected with siRNAs against HuR. GATA3 and its pre-mRNA but not KIAA1429 showed dramatic decreases upon HuR knockdown (Fig. 4h and Additional file 8: Figures S6 g-l). Our findings showed that HuR fortifies the stability of GATA3 pre-mRNA by binding to the 3′ UTR without KIAA1429-mediated m6A methylation.

### GATA3-AS functions as a guide lncRNA that promotes the m6A modification of GATA3 pre-mRNA by KIAA1429 in a targeted manner

**Fig. 5:**
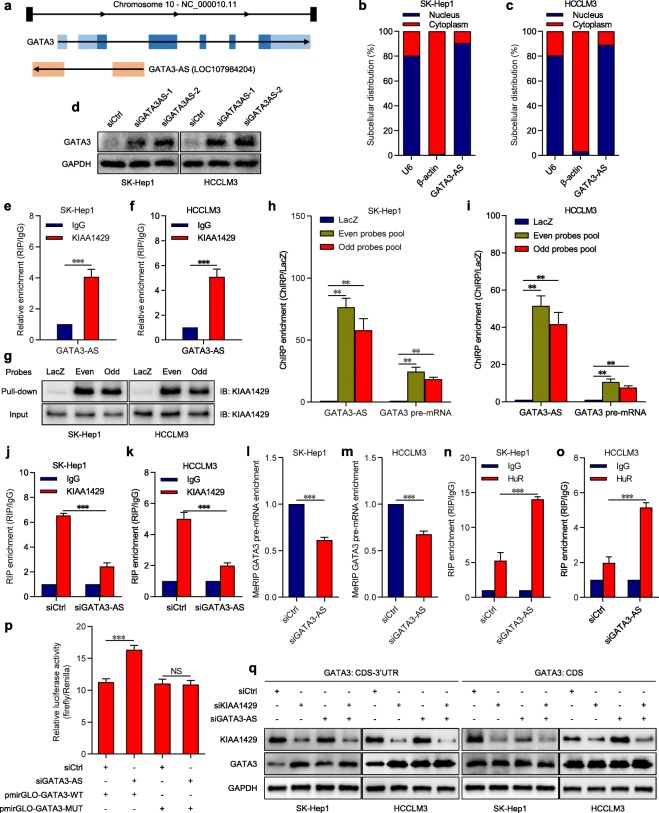
GATA3-AS functions as a guide lncRNA that targetedly promotes the m6A modification of GATA3 pre-mRNA. **a** Genomic organization of GATA3 and GATA3-AS transcription units. Vertical bars represent exons. Arrows display the direction of transcription. **b** and **c** RNA distribution analysis of GATA3-AS in subcellular fractions of SK-Hep1 and HCCLM3 cells assessed by qPCR. U6 served as the nuclear marker, β-actin served as cytoplasmic marker. **d** Western blot analysis of GATA3 expression in SK-Hep1 and HCCLM3 cells transfected with GATA3-AS siRNAs or the control. **e** and **f** RIP-seq of the enrichment of GATA3-AS on KIAA1429 relative to IgG. **g** Western blot analysis of KIAA1429 in protein samples pulled down by even and odd sets for GATA3-AS, and control LacZ probes pool in SK-Hep1 and HCCLM3 cells. **h** and **i** ChIRP-qPCR analysis of the enrichment of GATA3-AS and GATA3 pre-mRNA in both even and odd sets relative to control LacZ probes pool in SK-Hep1 and HCCLM3 cells. **j** and **k** RIP-qPCR analysis of the enrichment of GATA3 pre-mRNA on KIAA1429 relative to IgG in control and GATA3-AS-depleted cells. **l** and **m** MeRIP-qPCR analysis of GATA3 pre-mRNA in SK-Hep1 and HCCLM3 cells transfected with GATA3-AS siRNAs or the control. **n** and **o** RIP-qPCR analysis of the enrichment of GATA3 pre-mRNA on HuR relative to IgG in control and GATA3-AS-depleted cells. **p** Relative luciferase activity in HEK293T cells co-transfected with luciferase reporter pmirGLO-GATA3-WT or pmirGLO-GATA3-MUT and GATA3-AS siRNAs or the control. Data are shown as the relative ratio of firefly luciferase activity to renilla luciferase activity. **q** Western blot analysis of GATA3 expression in SK-Hep1 and HCCLM3 cells co-transfected with GATA3 CDS-3′ UTR or GATA3 CDS vector and KIAA1429 siRNAs, GATA3-AS siRNAs or the control. Data are presented as mean ± SEM. NS: not significant; **P < 0.01, ***P < 0.001

Accumulating evidence has indicated that m6A methylation is a global and dynamic process in human cells [25, 26]. The same RNA transcript may show different m6A levels under diverse physiological or pathological conditions, possibly due to certain *cis* or *trans* factors [27]. Hence, we speculated that there might be specific factors that contribute to the preferential recognition of KIAA1429 on GATA3 pre-mRNA in liver cancer. Additionally, recent studies reported that antisense transcript-derived long noncoding RNAs (lncRNAs) participate in the determination of heritable cell-specific alternative splicing of the parental pre-mRNAs by *cis* regulation [28, 29]. Accordingly, a local lncRNA, LOC107984204, located on chromosome 10 (chr10: 8054079–8,057,382, GRCh38.p13 Primary Assembly) came to our attention. Since this lncRNA is transcribed from the antisense strand of the GATA3 gene and has 653 nucleotides complementary to GATA3 pre-mRNA (Fig. 5a), we referred to it as GATA3-AS.

Next, we preliminarily analyzed the correlation between the expression of GATA3 pre-mRNA and GATA3-AS in clinical samples from the WCH dataset. The results showed that GATA3-AS was statistically related to GATA3 pre-mRNA (Additional file 8: Figure S6 m). Subcellular RNA fractionation assays showed that GATA3-AS was mainly localized in the nucleus (Figs. 5), which was in accordance with the subcellular location of GATA3 pre-mRNA. More importantly, depletion of GATA3-AS (Additional file 9: Figures S7a-b) led to significantly enhanced expression of GATA3 pre-mRNA (Additional file 9: Figures S7c-d) and then the remarkable rise of GATA3 at the RNA and protein levels (Fig. 5d and Additional file 9: Figures S7e-f). Based on the above findings and the sequence complementarity between GATA3-AS and GATA3 pre-mRNA, it can be deduced that GATA3-AS might serve as a *cis*-acting element involved in the targeted regulation of KIAA1429 on GATA3 pre-mRNA. To verify this hypothesis, we launched the combined application of RNA fluorescence in situ hybridization (FISH) and immunofluorescence (IF), which further confirmed the nuclear co-localization of GATA3-AS and KIAA1429 (Additional file 8: Figure S6n). Following that, RIP assays demonstrated that GATA3-AS was statistically enriched in KIAA1429 immunoprecipitates compared with the IgG pellet (Figs. 5e-f). In the opposite manner, we performed RNA pull-down assays by using biotin-labeled oligonucleotide probes targeting GATA3-AS, all of which were divided into an even probes pool and an odd probes pool for the purpose of improving the specificity [30]. The data demonstrated the tremendous enrichment of KIAA1429 in both the even and odd sets relative to the control LacZ probes pool (Fig. 5g), validating the interaction between GATA3-AS and KIAA1429. Nevertheless, GATA3-AS showed nonstatistical enrichment in HuR- immunoprecipitated RNAs and HuR could not be detected in either even or odd probes set (Additional file 9: Figures S7 g-i), which excluded the possibility that HuR could bind to GATA3-AS. Thereafter, ChIRP assays were implemented to identify the direct interaction between GATA3-AS and GATA3 pre-mRNA, illustrating that both the even and odd sets corresponding to GATA3-AS were associated with drastically higher enrichment of GATA3 pre-mRNA than the control LacZ probes pool (Figs. 5h-i). Overall, our observations suggested that GATA3-AS specifically interacts with KIAA1429 and GATA3 pre-mRNA.

To further explore whether GATA3-AS was required for the specific selection of GATA3 pre-mRNA by KIAA1429, we compared the enrichment of GATA3 pre-mRNA in KIAA1429 immunoprecipitates for control and GATA3-AS knockdown cells by using RIP assays, which indicated that GATA3-AS specifically facilitated the interaction between KIAA1429 and GATA3 pre-mRNA (Figs. 5j-k). Consistently, MeRIP-qPCR revealed that GATA3-AS inhibition significantly lowered the m6A level of GATA3 pre-mRNA (Figs. 5l-m), which reinforced the binding of HuR to GATA3 pre-mRNA (Figs. 5n-o) and enhanced the luciferase activity of pmirGLO-GATA3-WT rather than pmirGLO-GATA3-MUT (Fig. 5p). Eventually, we found that GATA3-AS exerted similar effects via pKIAA1429 on GATA3 expression in cells transfected with GATA3 CDS-3′ UTR or GATA3 CDS alone vector (Fig. 5q). By contrast, neither KIAA1429 nor GATA3-AS experienced any significant change after silencing GATA3 (Additional file 9: Figures S7j-m), and GATA3-AS did not accept the regulation from KIAA1429 (Additional file 9: Figures S7n-o). Taken together, our results showed that GATA3-AS guides KIAA1429 to preferentially mediate the m6A modification of GATA3 pre-mRNA by simultaneously interacting with KIAA1429 and GATA3 pre-mRNA.

In terms of the complexity of m6a modification, a biochemical study revealed that KIAA1429 recruits the catalytic core components METTL3/METTL14 to guide region-selective methylation [17]. Specifically, the activity of KIAA1429 is dependent on the METTL3/METT14 complex. We next examined the effects of METTL3 or METTL14 knockdown on the GATA3 m6A modification by using MeRIP-qPCR. The data demonstrated that upon silencing METTL3 or METTL14 (Additional file 10: Figures S8a-f), the m6A methylation level of GATA3 pre-mRNA presented a statistical decrease (Additional file 10: Figures S8 g-j) that was significantly less than the decrease observed upon knockdown of KIAA1429. Additionally, the expression of GATA3 pre-mRNA and mRNA displayed significant increases in METTL3 or METTL14-inhibited cells (Additional file 10: Figures S8k-r), although these increases were substantially lower than that in KIAA1429-inhibited cells. It can be seen that KIAA1429 was required for the full m6a methylation program, which was consistent with a previously reported study [12].

### GATA3 mediates the tumor growth and metastasis driven by KIAA1429 or GATA3-AS in vitro and in vivo

**Fig. 6:**
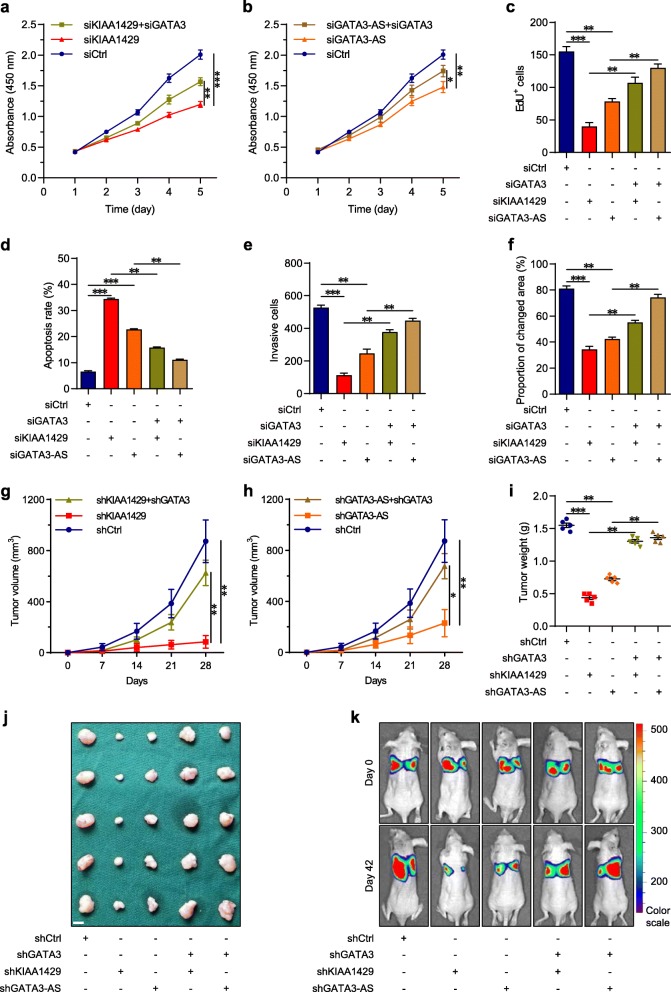
GATA3 mediates the cell proliferation and invasion driven by KIAA1429 or GATA3-AS. **a** and **b** CCK-8 assays for indicated cells. **c** EdU immunofluorescence staining assays for indicated cells. **d** Cell apoptosis was measured by FITC-Annexin V and PI staining in indicated cells, followed by flow cytometric analysis. **e** Transwell invasion assays for indicated cells. **f** Wound-healing migration assays for indicated cells. **g** and **j** Tumor volume and tumor weight in subcutaneous xenografts models with indicated cells. Scale bars = 5 mm. **k** Representative bioluminescence photographs of the murine tail vein injection lung metastasis models at indicated times by imaging with the IVIS@ Lumina II system. Data are presented as mean ± SEM. *P < 0.05, **P < 0.01, ***P < 0.001

**Fig. 7:**
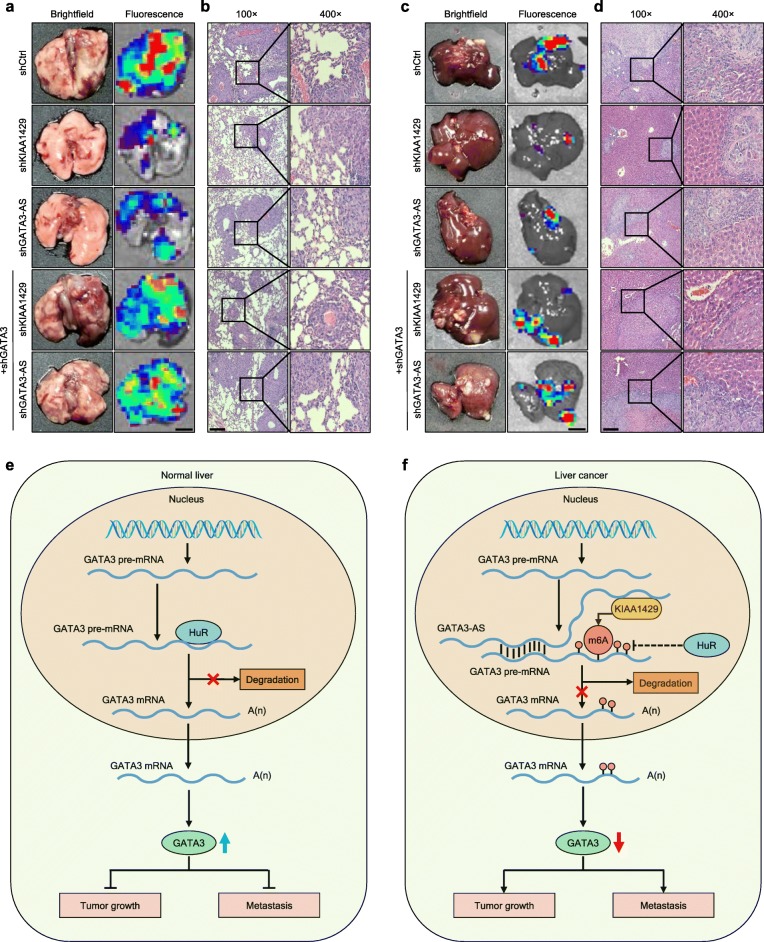
GATA3 mediates the metastasis driven by KIAA1429 or GATA3-AS. **a** Representative brightfield and fluorescence photographs of pulmonary metastatic nodules in lung metastasis models with indicated cells. Scale bars = 2 mm. **b** Representative microscopic views of pulmonary metastatic foci from indicated cells in tissue sections of lungs using HE staining. Scale bars = 100 μm. **c** Representative brightfield and fluorescence photographs of intrahepatic metastatic nodules in orthotopic implantation models with indicated cells. Scale bars = 5 mm. **d** Representative microscopic views of intrahepatic metastatic foci from indicated cells in tissue sections of livers using HE staining. Scale bars = 100 μm. **e** and **f** Schematic Model of the epi-transcriptomic regulation underlying the KIAA1429-GATA3 pathway. Data are presented as mean ± SEM

To confirm whether GATA3 was a dominant contributor to the malignant cell phenotypes promoted by KIAA1429 or GATA3-AS, we evaluated whether GATA3 inhibition could rescue the effects of KIAA1429 or GATA3-AS knockdown on the biological behaviors of hepatoma cells. Specifically, GATA3-AS or KIAA1429-inhibited cells were treated with siRNAs against GATA3. Depletion of GATA3 significantly reversed the influence of silencing GATA3-AS or KIAA1429 on cell proliferation, apoptosis resistance, invasion and migration in vitro (Figs. 6a-f and Additional file 11: Figures S9a-d). Thereafter, further rescue experiments in vivo were conducted. Stable GATA3-AS or KIAA1429-inhibited cells were effectively infected with LV-shGATA3. Although the volume and weight of subcutaneous tumors with LV-shKIAA1429 or LV-shGATA3-AS displayed considerable decreases, GATA3 suppression in these two groups reinstated tumor growth (Figs. 6g-j). Additionally, regarding the lung metastasis models, inhibiting GATA3 largely recovered the decreased bioluminescence signal intensities in mice (Fig. 6k), the decreased fluorescence signal intensities of lung metastatic nodules and the fewer pulmonary metastatic foci caused by GATA3-AS or KIAA1429 inhibition (Figs. 7a-b and Additional file 11: Figure S9e). Likewise, the impaired intrahepatic metastatic potential triggered by depleting GATA3-AS or KIAA1429 could be restored by GATA3 knockdown (Figs. 7c-d and Additional file 11: Figure S9f), showing that GATA3 mediated the oncogenic functions of KIAA1429 and GATA3-AS.

Eventually, we assessed the association between GATA3 expression and the clinical characteristics of 70 HCC samples from the WCH dataset, which illustrated that low expression of GATA3 was significantly correlated with microvascular invasion (*P* = 0.0168), TNM stage (*P* = 0.0220) and BCLC stage (*P* = 0.0220) (Additional file 12: Table S2). Moreover, decreased GATA3 expression was statistically related to poor overall survival and disease-free survival (Additional file 11: Figures S9 g-h). The univariate and multivariate Cox regression analysis identified GATA3 as an independent risk factor for prognosis (Additional file 13: Table S3, Additional file 14: Table S4, Additional file 15: Table S5 and Additional file 16: Table S6). More strikingly, we observed that patients with low KIAA1429 and high GATA3 expression showed greatly improved overall survival and disease-free survival than others (Additional file 11: Figures S9i-j). Collectively, these findings suggested that GATA3 could act as a critical prognostic biomarker for HCC patients.

## Discussion

As the predominant facilitators of all vital activities, proteins are generated by the translation of RNA transcribed from DNA. RNA is in charge of the genetic transinformation. At present, chemical modifications on DNA and protein, such as methylation, phosphorylation and acetylation, have been explored in-depth [31, 32]. RNA also can undergo more than 100 distinct chemical modifications [33]. Nonetheless, the popularity, functions and mechanisms of diverse RNA modifications are still largely undiscovered, among which m6A methylation is the most common modification on eukaryotic RNA. An increasing number of studies have addressed the pathological significance of m6A dysregulation in human diseases, especially in cancers [34, 35]. For m6A writers, the specific functions and molecular mechanisms of METTL3, METTL14 and WTAP have been extensively studied in various malignancies [5, 35, 36]. The role of KIAA1429 in carcinogenesis and metastasis remains largely unknown. A recent report described the upregulation of KIAA1429 in HCC tissues from The Cancer Genome Atlas (TCGA) dataset [37]. Accordingly, our data identified KIAA1429 as a formidable driver of liver tumor growth and metastasis. Specifically, upregulation of KIAA1429 was observed in 70 HCC tissues and was significantly associated with the clinical characteristics and prognosis of HCC patients. Additionally, KIAA1429 remarkably induced the cell cycle, proliferation, apoptosis resistance, invasion and migration in vitro and facilitated tumor growth, pulmonary and intrahepatic metastasis in vivo, which confirmed the tumorigenic and metastasis-promoting functions of KIAA1429.

As the largest known component in the m6A methyltransferase complex, KIAA1429 is identified as a scaffold that orchestrates the core components, which consist of METTL3/METTL14/WTAP, on RNA substrates for the purpose of specific m6A methylation in the 3′ UTR and near the stop codon [17, 38]. Indeed, we found significantly lower global m6A levels in KIAA1429-inhibited cells, which highlighted the functional importance of KIAA1429 in m6A modification. Therefore, MeRIP-seq was performed to clarify the altered m6A peaks on methylated transcripts caused by KIAA1429 depletion, consistently verifying 1152 decreased peaks in the 3′ UTR and near the stop codon. Strikingly, it is reported that KIAA1429 contains a RNA-binding protein (RBP) domain similar to those of RNA and DNA helicases, translation initiation factors and ribonucleoproteins, which is involved in RNA metabolism, processing, transport and translation [39]. Research has proven that abnormal expression of certain RBPs may be the main reason for the activation of oncogenes or the downregulation of tumor suppressor genes [40], which prompted us to conduct RIP-seq to probe the decisive tumor-related transcripts bound by KIAA1429. Then, RNA-seq was employed to ascertain the altered expression of mRNA transcripts modulated by KIAA1429. Eventually, we identified GATA3 as the direct downstream target of KIAA1429-mediated m6A modification.

Hundreds of reports describe the cancer-inhibiting implications of GATA3 [41]. As a well-known transcription factor, GATA3 induces the expression of numerous tumor-suppressive genes by binding to and activating their promoters [21, 42]. However, limited research has focused on what causes the downregulation of GATA3 in cancers. In our work, KIAA1429 was found to mediate the m6A modification on the 3′ UTR of GATA3 pre-mRNA, disturbing the binding of HuR to GATA3 pre-mRNA and then reducing the expression of GATA3 pre-mRNA, after which GATA3 mRNA displayed decreased expression. Furthermore, we noted that silencing GATA3 exerted reversed effects on cell proliferation and metastasis restrained by KIAA1429 depletion. In fact, this is first comprehensive work that investigates the biological function of GATA3 in HCC and explains why GATA3 is down-regulated.

LncRNAs are a class of RNA transcripts that contain more than 200 nucleotides and have no protein-coding potential [43]. As a subtype of lncRNAs, antisense transcript-derived lncRNAs play crucial roles in gene regulation [29]. For example, the antisense lncRNA FGFR2-AS from within the FGFR2 locus promotes function-specific alternative splicing of FGFR2 pre-mRNA [28]. Antisense lncRNA of glutaminase (GLS-AS) inhibits GLS expression at the post-transcriptional level via interaction with GLS pre-mRNA in pancreatic cancer [44]. In this study, we identified GATA3-AS, transcribed from the antisense strand of the GATA3 gene, as a *cis*-acting element for targeted m6A regulation of KIAA1429 on GATA3 pre-mRNA. In addition, our data revealed that GATA3-AS knockdown markedly suppressed the malignant phenotypes of hepatoma cells, which could be rescued by GATA3 inhibition.

## Conclusions

In conclusion, we ascertained a complex epi-transcriptomic regulation underlying the KIAA1429-GATA3 pathway. To be precise, without KIAA1429 disturbance, the interaction between HuR and GATA3 pre-mRNA sustained the stability of the latter in normal liver cells, leading to a relatively high expression of GATA3 (Fig. 7e). In contrast, under the guidance of GATA3-AS, KIAA1429 preferentially induced m6A methylation on the 3′ UTR of GATA3 pre-mRNA in liver cancer cells, followed by the separation of HuR and the degradation of GATA3 pre-mRNA, before GATA3 showed downregulated expression (Fig. 7f). In conclusion, our findings contributed insight into the critical role of KIAA1429 in liver cancer development, and indicated the novel significance of the molecular mechanism of m6A epi-transcriptomic modification in cancer research.

## Supplementary information

**Additional file 1.** Additional Materials and Methods.

**Additional file 2: Figure S1.** KIAA1429 is elevated in HCC tissues. a, KIAA1429 expression in 50 pairs of HCC tissues and adjacent normal tissues from TCGA dataset. b-c, Kaplan-Meier analyses of the correlations between KIAA1429 expression and overall survival or disease-free survival of 50 HCC patients. d-e, ROC analysis of KIAA1429 expression in HCC tissues and adjacent normal tissues from TCGA and WCH datasets. f, Representative IHC stains of KIAA1429 in HCC tissues and adjacent normal tissues. g, Western blot analysis of GATA3 expression in 5 pairs of HCC tissues and adjacent normal tissues. h, GATA3 expression in seven human hepatoma cell lines. Data are presented as mean ± SEM.

**Additional file 3: Table S1.** Clinical characteristics of 70 HCC patients according to KIAA1429 expression level.

**Additional file 4: Figure S2.** Inhibition of KIAA1429 impairs the cell proliferation and migration in vitro. a-b, GATA3 expression in SK-Hep1 and HCCLM3 cells transfected with KIAA1429 siRNAs or the control by using qPCR. c, Western blot analysis of GATA3 expression in SK-Hep1 and HCCLM3 cells transfected with KIAA1429 siRNAs or the control. d-e, CCK-8 assays for SK-Hep1 and HCCLM3 cells transfected with KIAA1429 siRNAs or the control. f-g, Cell cycle distribution was measured by PI staining in SK-Hep1 and HCCLM3 cells transfected with KIAA1429 siRNAs or the control, followed by flow cytometric analysis. h-i, Wound-healing migration assays for SK-Hep1 and HCCLM3 cells transfected with KIAA1429 siRNAs or the control. Scale bars = 100 μm. Data are presented as mean ± SEM. **P < 0.01.

**Additional file 5: Figure S3.** Overexpression of KIAA1429 promotes the cell proliferation and metastasis in vitro. a-b, Fluoresence signal intensities of livers in each group after orthotopic implantation with indicated SK-Hep1 and HCCLM3 cells. c-d, Fluoresence signal intensities of lungs in each group after tail intravenous injection with indicated SK-Hep1 and HCCLM3 cells. e-f, CCK-8 assays for Huh-7 and SNU-182 cells with or without KIAA1429 upregulation. g-h, Cell cycle distribution was measured by PI staining in Huh-7 and SNU-182 cells with or without KIAA1429 upregulation, followed by flow cytometric analysis. i-j, EdU immunofluorescence staining assays for Huh-7 and SNU-182 cells with or without KIAA1429 upregulation. Scale bars = 100 μm. k-l, Cell apoptosis was measured by FITC-Annexin V and PI staining in Huh-7 and SNU-182 cells with or without KIAA1429 upregulation, followed by flow cytometric analysis. m-n, Wound-healing migration assays for Huh-7 and SNU-182 cells with or without KIAA1429 upregulation. Scale bars = 100 μm. Data are presented as mean ± SEM. NS: not significant; **P < 0.01, ***P < 0.001.

**Additional file 6: Figure S4.** Analysis of differentially expressed genes by KIAA1429 knockdown in RNA-seq. a-b, Transwell invasion assays for Huh-7 and SNU-182 cells with or without KIAA1429 upregulation. Scale bars = 100 μm. c-d, Gene Ontology and KEGG pathway analysis of the differentially expressed genes by KIAA1429 knockdown in RNA-seq. Data are presented as mean ± SEM. ***P < 0.001.

**Additional file 7: Figure S5.** KIAA1429 mediates the m6A modification of GATA3 pre-mRNA. a-b, The RNA levels of GATA3 mRNA at the indicated time points were analyzed by qPCR relative to time 0 after blocking new RNA synthesis with actinomycin D (1 mg/mL) in SK-Hep1 and HCCLM3 cells and normalized to 18S rRNA. c-d, Relative luciferase activity of the GATA3 promoter firefly luciferase reporter in SK-Hep1 and HCCLM3 cells transfected with KIAA1429 siRNAs or the control. Data are shown as the relative ratio of firefly luciferase activity to renilla luciferase activity. e-f, GATA3 pre-mRNA expression in SK-Hep1 and HCCLM3 cells transfected with KIAA1429 siRNAs or the control. g-h, RNA distribution analysis of GATA3 mRNA and GATA3 pre-mRNA in subcellular fractions of SK-Hep1 and HCCLM3 cells assessed by qPCR. U6 served as the nuclear marker, β-actin served as cytoplasmic marker. i, Protein distribution analysis of KIAA1429 in subcellular fractions of SK-Hep1 and HCCLM3 cells assessed by western blot. Histone H3 served as the nuclear marker, β-Tubulin served as cytoplasmic marker. j-k, RIP-seq of the enrichment of GATA3 pre-mRNA on KIAA1429 relative to IgG. l-m, MeRIP-qPCR analysis of GATA3 pre-mRNA in SK-Hep1 and HCCLM3 cells transfected with KIAA1429 siRNAs or the control. Data are presented as mean ± SEM. NS: not significant; *P < 0.05, ***P< 0.001.

**Additional file 8: Figure S6.** HuR mediates the regulation of GATA3 by KIAA1429. a-b, GATA3 expression in SK-Hep1 and HCCLM3 cells co-transfected with GATA3 CDS-3’ UTR or GATA3 CDS vector and KIAA1429 siRNAs or the control by using qPCR. c-d, HuR expression in SK-Hep1 and HCCLM3 cells transfected with HuR siRNAs or the control by using qPCR. e-f, RIP-seq of the enrichment of GATA3 mRNA on HuR relative to IgG. g-h, GATA3 pre-mRNA expression in SK-Hep1 and HCCLM3 cells transfected with HuR siRNAs or the control by using qPCR. i-j, GATA3 mRNA expression in SK-Hep1 and HCCLM3 cells transfected with HuR siRNAs or the control by using qPCR. k-l, KIAA1429 expression in SK-Hep1 and HCCLM3 cells transfected with HuR siRNAs or the control by using qPCR. m, Scatter plots of GATA3-AS versus GATA3 pre-mRNA expression in WCH data repository. Pearson correlation coefficients (r) and P values are shown. n, Combined application of FISH and IF. LNA probes for GATA3-AS (red) and a fluorescence-conjugatedsecondary antibody was used for KIAA1429 (green). Data are presented as mean ± SEM. NS: not significant; **P < 0.01, ***P < 0.001.

**Additional file 9: Figure S7.** GATA3-AS functions as a guide lncRNA that targetedly promotes the interaction of KIAA1429 with GATA3 pre-mRNA. a-b, GATA3-AS expression in SK-Hep1 and HCCLM3 cells transfected with GATA3-AS siRNAs or the control by using qPCR. c-d, GATA3 pre-mRNA expression in SK-Hep1 and HCCLM3 cells transfected with GATA3-AS siRNAs or the control by using qPCR. e-f, GATA3 mRNA expression in SK-Hep1 and HCCLM3 cells transfected with GATA3-AS siRNAs or the control by using qPCR. g-h, RIP-seq of the enrichment of GATA3-AS on HuR relative to IgG. i, Western blot analysis of HuR in protein samples pulled down by even and odd sets for GATA3-AS, and control LacZ probes pool in SK-Hep1 and HCCLM3 cells. j-k, KIAA1429 expression in SK-Hep1 and HCCLM3 cells transfected with GATA3 siRNAs or the control by using qPCR. l-m, GATA3-AS expression in SK-Hep1 and HCCLM3 cells transfected with GATA3 siRNAs or the control by using qPCR. n-o, GATA3-AS expression in SK-Hep1 and HCCLM3 cells transfected with KIAA1429 siRNAs or the control by using qPCR. Data are presented as mean ± SEM. NS: not significant; **P < 0.01, ***P < 0.001.

**Additional file 10: Figure S8.** Knockdown of METTL3 or METTL14 regulates the m6a modification and expression levels of GATA3. a-d, METTL3 or METTL14 expression in SK-Hep1 and HCCLM3 cells transfected with METTL3 or METTL14 siRNAs or the control by using qPCR. e-f, Western blot analysis of METTL3 or METTL14 expression in SK-Hep1 and HCCLM3 cells with METTL3 or METTL14 siRNAs or the control. g-j, MeRIP-qPCR analysis of GATA3 pre-mRNA in SK-Hep1 and HCCLM3 cells transfected with METTL3 or METTL14 siRNAs or the control. k-r, GATA3 pre-mRNA or mRNA expression in SK-Hep1 and HCCLM3 cells transfected with METTL3 or METTL14 siRNAs or the control by using qPCR. Data are presented as mean ± SEM. **P < 0.01, ***P < 0.001.

**Additional file 11: Figure S9.** GATA3 mediates the cell proliferation and metastasis driven by KIAA1429 or GATA3-AS in vitro. a, EdU immunofluorescence staining assays for indicated cells. Scale bars = 100 μm. b, Cell apoptosis was measured by FITC-Annexin V and PI staining in indicated cells, followed by flow cytometric analysis. c, Transwell invasion assays for indicated cells. Scale bars = 100 μm. d, Wound-healing migration assays for indicated cells. Scale bars = 100 μm. e, Fluoresence signal intensities of livers in each group after orthotopic implantation with indicated cells. f, Fluoresence signal intensities of lungs in each group after tail intravenous injection with indicated cells. g-h, Kaplan-Meier analyses of the correlations between GATA3 expression and overall survival or disease-free survival of 70 HCC patients. The median expression level was used as the cutoff. Values are expressed as the median with interquartile range. i-j, Kaplan-Meier analyses of the overall survival or disease-free survival between patients with low KIAA1429 and high GATA3 expressions and others. Data are presented as mean ± SEM. **P < 0.01.

**Additional file 12: Table S2.** Clinical characteristics of 70 HCC patients according to GATA3 expression level.

**Additional file 13: Table S3.** Univariate analysis of several variables for OS.

**Additional file 14: Table S4.** Multivariate analysis of several variables for OS.

**Additional file 15: Table S5.** Univariate analysis of several variables for DFS.

**Additional file 16: Table S6.** Multivariate analysis of several variables for DFS.

**Additional file 17.** Additional file legends.

## Data Availability

All data generated or analyzed during this study are included either in this article or in the supplementary information files. The RNA-seq, RIP-seq and MeRIP-seq datasets were deposited in Gene Expression Omnibus (GEO) database (https://www.ncbi.nlm.nih.gov/geo/), accession numbers: GSE134630, GSE134978 and GSE134776.

## Abbreviations

3′ UTR: 3′ untranslated region
ALKBH5: a-ketoglutarate-dependent dioxygenase alkB homolog 5
AS: antisense
CDS: coding sequences
ChIRP: chromatin isolation by RNA purification
FTO: fat-mass and obesity-associated protein
GATA3: GATA Binding Protein 3
HCC: hepatocellular carcinoma
HE: Hematoxylin and eosin
HuR: Hu-Antigen R
IF: immunofluorescence
IHC: immunohistochemical
LNA-FISH: locked nucleic acid-fluorescence in situ hybridization
lncRNA: long noncoding RNA
m6A: N6-methyladenosine
MeRIP-seq: methylated RNA immunoprecipitation sequencing
METTL14: methyltransferase like 14
METTL3: methyltransferase like 3
pre-mRNA: precursor messenger RNA
qPCR: quantitative PCR
RIP-seq: RNA immunoprecipitation sequencing
RNA-seq: RNA sequencing
siRNA: small interference RNA
STR: short tandem repeat
TCGA: The Cancer Genome Atlas
WCH: West China Hospital
WTAP: WT1 associated protein
YTHDF: YTH N6-methyladenosine RNA binding protein
