# Supplementary material for: KIAA1429 contributes to liver cancer progression through N6-methyladenosine-dependent post-transcriptional modification of GATA3
Source: Mol Cancer. 2019 Dec 19;18:186. doi: 10.1186/s12943-019-1106-z (PMC6921542; doi:10.1186/s12943-019-1106-z)
Supplement: Supplementary file 1 — Additional file 1. Additional Materials and Methods. [file 12943_2019_1106_MOESM1_ESM.docx]

**Additional Materials and Methods**

**TCGA LIHC data repository**

High-throughput RNA-sequencing data and whole-genome methylation data of HCC were downloaded from The Cancer Genome Atlas (TCGA) Liver Hepatocellular Carcinoma (LIHC) dataset [1]. The sequencing data of 370 HCC samples and 50 adjacent non-cancerous samples from Illumina HiSeq platform were analyzed in accordance with the TCGA publication guidelines (<http://cancergenome.nih.gov/>).

**RNA extraction and real-time quantitative PCR**

Total RNA was extracted by using Trizol reagent (Invitrogen, CA, USA). First-strand cDNA was synthesized by using the PrimeScript™ Reverse Transcriptase kit (Takara, Shiga, Japan) with random primers. Real-time quantitative PCR was performed with a ChamQ^TM^ SYBR^@^ qPCR Master Mix (Vazyme Biotech, Nanjing, China). All reactions were run in triplicate on the CFX96^TM^ Touch Real-Time PCR System (Bio-Rad, California, USA). GAPDH or U6 were applied as endogenous control. The relative expression was calculated using the comparative Ct (2^-ΔΔ^CT) method [2].

**Primers used in this study:**

| Primer names | Sequences |
| --- | --- |
| GAPDH forward | 5’-GGAGCGAGATCCCTCCAAAAT-3’ |
| GAPDH reverse | 5’-GGCTGTTGTCATACTTCTCATGG-3’ |
| KIAA1429 forward | 5'-CTTGGCAAGTGGCTTGAACC-3' |
| KIAA1429 reverse | 5'-ACGTAAGGCAGTGGTAAGGC-3' |
| METTL3 forward | 5'-ATCCCCAAGGCTTCAACCAG-3' |
| METTL3 reverse | 5'-GCGAGTGCCAGGAGATAGTC-3' |
| METTL14 forward | 5'-AGAGAACAAAGGAACACTGCCT-3' |
| METTL14 reverse | 5'-AATGAAGTCCCCGTCTGTGC-3' |
| GATA3 forward | 5'-CCTGCAGTCCCTTTCGACTT-3' |
| GATA3 reverse | 5'-GAGCTCCTTTGCAAAGTGGC-3' |
| GATA3-AS forward | 5'-AGCCTGCCCCATTCATGAAG-3' |
| GATA3-AS reverse | 5'-CTCTCCCAAACACCCTGCAT-3' |
| GATA3 pre-mRNA forward | 5’-CCATGCTGACCATTCTGGGT-3’ |
| GATA3 pre-mRNA reverse | 5’-AAAACTGTCCCAAGCCAGCT-3’ |
| m6A peak 1 forward | 5’-AACGCAATCTGACCGAGCA-3’ |
| m6A peak 1 reverse | 5’-GTCACCTGGGTAGCGAAGAG-3’ |
| m6A peak 2 forward | 5’-CCTGCGGGCTCTATCACAAA-3’ |
| m6A peak 2 reverse | 5’-TCTGACAGTTCGCACAGGAC-3’ |
| m6A peak 3 forward | 5’-CCTGCAGTCCCTTTCGACTT-3’ |
| m6A peak 3 reverse | 5’-GAGCTCCTTTGCAAAGTGGC-3’ |
| m6A peak 4 forward | 5’-CATCTGGGTAGCTGTAAGGCA-3’ |
| m6A peak 4 reverse | 5’-CTGTGGCCAGTGAAAGGAAAC-3’ |
| HuR forward | 5’-GGGTGACATCGGGAGAACGAAT-3’ |
| HuR reverse | 5’-TGTCCTGCTACTTTATCCCGAA-3’ |
| 18S rRNA forward | 5’-ACACGGACAGGATTGACAGA-3’ |
| 18S rRNA reverse | 5’-GGACATCTAAGGGCATCACA-3’ |
| U6 snRNA forward | 5’-GCTTCGGCAGCACATATACTAAAAT-3’ |
| U6 snRNA reverse | 5’-CGCTTCACGAATTTGCGTGTCAT-3’ |

**MeRIP-qPCR**

m6A methylation levels of specific RNAs were determined using Magna MeRIP^TM^ m6A Kit (Merck Millipore, Darmstadt, Germany). Briefly, 300 μg of total RNA treated with gDNA wiper mix (Vazyme, Nanjing, China) and the concentration was adjusted to 1µg/µl with nuclease-free water. Chemically fragmented RNA (100-nt) was immunoprecipitated with anti-m6A antibody (Synaptic Systems, Goettingen, Germany) according to the manufacturer’s instructions, and 1/10 of the fragmented RNA was saved as input control. m6A enrichment, normalized to input, in each sample was calculated by qPCR.

**Immunohistochemistry (IHC)**

For immunohistochemical (IHC) analysis, HCC specimens tissue slides were deparaffinized, rehydrated through an alcohol series followed by antigen retrieval with sodium citrate buffer. Tumor sections were blocked with 5% normal goat serum with 0.1% Triton X-100 and 3% H_2_O_2_ in PBS for 60 min at room temperature and then incubated with antibody against KIAA1429 (Novus Biologicals, CO, USA) overnight at 4°C. IHC staining was performed with horseradish peroxidase (HRP) conjugates using DAB detection. The KIAA1429 immunostaining degree was calculated as the sum of the score for the proportion of positively stained tumor cells (PP) and the score for staining intensity (SI). PP was scored into four categories: 0 (< 5%, negative), 1(5-25%, sporadic), 2 (25-50%, focal), 3 (> 51%, diffuse) and SI was scored on a scale of 0 to 3 (0, negative staining; 1, weak staining; 2, moderate staining; 3,strong staining). The final staining score was calculated by multiplying SI and PP score, resulting in a score value ranging from 0 to 9. The positive level of IHC staining was scored by two independent pathologists who were blinded to the clinical characteristics and the subgroups of the patients. When there was a disagreement about scoring, a consensus was reached by discussion. Staining results were classified as follows: negative (0); low positive (1-3); medium positive (4-6); high positive (7-9).

**Immunofluorescence (IF)**

For immunofluorescence (IF) analysis, cultured cells were fixed with 4% formaldehyde for 20 min and then blocked with 5% normal goat serum with or without 0.1% Triton X-100 in PBS for 60 min at room temperature. Immunostaining was performed using antibody against KIAA1429 (Novus Biologicals, CO, USA) and Alexa Fluor 488 secondary antibody (Invitrogen, CA, USA). Nuclei were counterstained with Hoechst. Fluorescence signals were scanned using the A1R+MP confocal laser microscope system (Nikon, Tokyo, Japan).

**RNA fluorescence in situ hybridization (FISH)**

Locked nucleic acid-fluorescence in situ hybridization (LNA-FISH) was performed to determine the subcellular location of GATA3-AS. LNA fluorescein-labeled probes against GATA3-AS were designed and synthesized by RiboBio (RiboBio Biotechnology, Guangzhou, China). FISH was conducted using the Fluorescent in Situ Hybridization Kit (RiboBio Biotechnology, Guangzhou, China), according to the manufacturer’s protocol. Fluorescence signals were scanned using the A1R+MP confocal laser microscope system (Nikon, Tokyo, Japan).

**RNA immunoprecipitation (RIP)**

RIP was performed using the Magna RIP^TM^ RNA-binding Protein Immunoprecipitation Kit (Millipore, Massachusetts, USA), according to the manufacturer’s protocol. Briefly, magnetic beads coated with 10 ug of specific antibodies against KIAA1429 (Cell Signaling Technology, Boston, USA), HuR (Abcam, Cambridge, USA) or normal IgG (Millipore, Massachusetts, USA) were incubated with prepared indicated cell lysates overnight at 4°C. Washed RNA-protein complexes were treated with proteinase K digestion buffer. The coprecipitated RNAs were purified with phenol: chloroform: isoamyl alcohol and subsequenty analyzed by qPCR to assess the enrichment of GATA3 pre-mRNA and GATA3-AS to KIAA1429 or HuR.

**Chromatin isolation by RNA purification (ChIRP) and RNA Pull-down**

Eight oligonucleotide probes corresponding to the GATA3-AS transcript were synthesized with biotin tags located at the 3’ end by RiboBio (RiboBio Biotechnology, Guangzhou, China). To eliminate nonspecific signals, all probes were divided into two pools (even and odd probe sets). The probe set targeting LacZ was used as a negative control. ChIRP assays were conducted using the EZ-Magna ChIRP RNA Interactome Kit (Millipore, Massachusetts, USA), according to the manufacturer’s protocol. 1/4 associated RNA-protein complexes were separated for RNA purification, and the remaining were subjected to protein purification. After being washed 6 times, the samples for RNA purification were subjected to proteinase K digestion and RNA extraction by TRIzol. The purified RNA was identified and quantified by qPCR to analyze the enrichment of GATA3 pre-mRNA to GATA3-AS. On the other hand, 3/4 associated RNA-protein complexes were treated with Elution Buffer and successively subjected to RNase A and RNase H digestion, and DNase digestion. The purified proteins were separated on 10% SDS-PAGE gels and analyzed by Western blot.

**Sequences of probes used for ChIRP in this study:**

| Probe names | Sequences |
| --- | --- |
| GATA3-AS ChIRP-Probe-1 | 5’-CGGTAGAGGCAGACTTAATA/3bio-TEG/-3’ |
| GATA3-AS ChIRP-Probe-2 | 5’-CCTTGACACGGATGACATAG/3bio-TEG/-3’ |
| GATA3-AS ChIRP-Probe-3 | 5’-TAATAATGATCCATGCGTGC/3bio-TEG/-3’ |
| GATA3-AS ChIRP-Probe-4 | 5’-CTTTATCCGATGACTCACCC/3bio-TEG/-3’ |
| GATA3-AS ChIRP-Probe-5 | 5’-TCAAGAAGTGTGTGGATTTG/3bio-TEG/-3’ |
| GATA3-AS ChIRP-Probe-6 | 5’-TAGAACCTCCTTAAGTTGCG/3bio-TEG/-3’ |
| GATA3-AS ChIRP-Probe-7 | 5’-CCCTTCCTCTCCCTTAAGAT/3bio-TEG/-3’ |
| GATA3-AS ChIRP-Probe-8 | 5’-CCACATTTAAAGGGCCAGAG/3bio-TEG/-3’ |

**RNA interference and production of lentiviral particles**

The small interfering RNA (siRNA) oligonucleotides against KIAA1429, GATA3-AS, GATA3, HuR and control siRNA were synthesized by PEPTBIO (PEPTBIO, Wuhan, China). The human KIAA1429 sequence was synthesized and cloned into the ORF lentiviral expression vector pReceiver-Lv206 (GeneCopoeia, CA, USA), named pReceiver-Lv206-KIAA1429. For knockdown of KIAA1429, GATA3-AS and GATA3, the same cDNA oligonucleotides with KIAA1429 siRNA-1, GATA3-AS siRNA-1 and GATA3 siRNA-1 were synthesized, respectively. With annealing, double-strand oligonucleotides were inserted into shRNA expression plasmid psi-LVRU6GP (GeneCopoeia, CA, USA). The lentiviral luciferase vector was purchased from GeneCopoeia. Viruses were produced in HEK-293T cells as previous described [3].

**Sequences of siRNA against specific target in this study:**

| Primer names | Sequences |
| --- | --- |
| siKIAA1429-1 sense | 5’-CCAUCAUCUUUAGACCUAATT-3’ |
| siKIAA1429-1 anti-sense | 5’-UUAGGUCUAAAGAUGAUGGTT-3’ |
| siKIAA1429-2 sense | 5'-CCUUACGUGUUCUCUGUAATT-3' |
| siKIAA1429-2 anti-sense | 5'-UUACAGAGAACACGUAAGGTT-3' |
| siGATA3AS-1 sense | 5'-GCAACUUAAGGAGGUUCUATT-3' |
| siGATA3AS-1 anti-sense | 5'-UAGAACCUCCUUAAGUUGCTT-3' |
| siGATA3AS-2 sense | 5’-GGAUCUAAUGCAGGGUGUUTT-3’ |
| siGATA3AS-2 anti-sense | 5’-AACACCCUGCAUUAGAUCCTT-3’ |
| siGATA3-1 sense | 5’-UCUGCUUCAUGGAUCCCUATT-3’ |
| siGATA3-1 anti-sense | 5’-UAGGGAUCCAUGAAUCAGATT-3’ |
| siGATA3-2 sense | 5’-GGGCUCUACUACAAGCUUCTT-3’ |
| siGATA3-2 anti-sense | 5’-GAAGCUUGUAGUAGAGCCCTT-3’ |
| siHuR-1 sense | 5’-GGUUUGGCUUUGUGACCAUTT-3’ |
| siHuR-1 anti-sense | 5’-AUGGUCACAAAGCCAAACCTT-3’ |
| siHuR-2 sense | 5'-GAACGAAUUUGAUCGUCAATT-3' |
| siHuR-2 anti-sense | 5'-UUGACGAUCAAAUUCGUUCTT-3' |
| Control siRNA sense | 5'-UUCUCCGAACGUGUCACGUTT-3' |
| Control siRNA anti-sense | 5'-ACGUGACACGUUCGGAGAATT-3' |

***In vitro* cell proliferation, cell cycle and cell apoptosis assays**

Cells were seeded in 96-well plates at a density of 2,000 cells/well and allowed to grow for five consecutive days. 10 μl of Cell Counting Kit-8 (CCK-8) (Beyotime Biotechnology, Shanghai, China) was added to each well, followed by incubation at 37°C for 1.5 h during the five days. The absorbance of each well was calculated at a wavelength of 450 nm using the Eon^TM^ Microplate Reader (BioTek, VT, USA). EdU (5-Ethynyl-2’-deoxyuridine) labelling assays were performed to assess the cell proliferation as well. Cells were seeds in 24-well plates at a density of 20,000 cells/well. 50 μM EdU (RiboBio Biotechnology, Guangzhou, China) was added to each well for 2 h, after which cells were fixed with 4% paraformaldehyde and quenched in glycine solution, followed by incubation with Apollo dye solution for 30 min. Images were captured using the OBSERVER D1/AX10 cam HRC microscope (Zeiss, Oberkochen, Germany) with a charge-coupled device (CCD) camera. Cell cycle and cell apoptosis assays were conducted based on flow cytometric analysis by using Cell Cycle and Apoptosis Kit (4A Biotech, Beijing, China), according to manufacturer’s instructions. Data were recorded and analyzed by the CytoFLEX Research Flow Cytometer (Beckman Coulter, CA, USA).

***In vitro* invasion and migration assays**

Transwell chambers were put into 24-well plates and the permeable 8.0 μm polycarbonate membranes were precoated with Matrigel. DMEM medium containing 10% FBS was added to the lower chamber, whereas 20,000 cells were plated in the upper chamber. After incubation at 37°C for 24 h, cells that invaded through the filters were fixed with 4% paraformaldehyde and stained with 0.1% crystal violet. The number of cells was counted in five distinct regions using the OBSERVER D1/AX10 cam HRC microscope (Zeiss, Oberkochen, Germany). For the migration assays, cells were seeded in 6-well plates at a density of 1 ×10^6^ cells/well. Confluent cell monolayers was destroyed by standardized wound scratching using a sterile 10 μl pipette tip and incubated in serum-free DMEM medium. After 24/48 h of wound formation, the proportion of changed area was calculated by Image J (National Institutes of Health, USA).

***In vivo* tumourigenesis and metastasis assays**

The animal studies were authorized by the Animal Ethic Review Committees of the West China Hospital. Male athymic BALB/c nude mice, aged 4-5 weeks, were purchased from BEIJING HFK BIOSCIENCE (Beijing, China) and were fed under standard pathogen-free conditions. Cells (5 × 10^5^) were injected subcutaneously into the right axillas of mice. Tumor growth was measured weekly with a caliper and tumor volume was calculated as length × width^2^ × 0.52. The mice were euthanized after 4 weeks and tumor weight was measured with an electronic scale. Tail intravenous injection models were established for lung metastasis assays. After 6 weeks, the metastases were visualized using the IVIS@ Lumina II system (Caliper Life Sciences, Hopkinton, MA) 15 min after intraperitoneal injection of 3.0 mg of D-Luciferin (ABP Biosicences, USA) in 200 µl of sterile PBS without magnesium or calcium. Furthermore, 20 μl Matrigel containing 1 × 10^6^ cells were injected into the livers of mice to construct orthotopic implanted models for liver metastasis assays. Mice were anatomized after 6 weeks and metastases were visualized using the IVIS@ Lumina II system (Caliper Life Sciences, Hopkinton, MA). The number of metastatic foci was counted in H&E staining in tissue sections of lungs and livers under the AX10 imager A2/AX10 cam HRC microscope (Zeiss, Oberkochen, Germany). All animal experiments were strictly implemented in compliance with the NIH Guide for the Care and Use of Laboratory Animals.

**Construction of luciferase reporter plasmids**

The promoter region of GATA3 was synthesized and subcloned into the pEZX-PL01 vector (GeneCopoeia, CA, USA). Wild-type pmirGLO-GATA3-WT (Promega, Madison, WI) reporter plasmid was cloned by inserting the 3’ UTR of GATA3 transcript after the Fluc coding sequence. The mutant pmirGLO-GATA3-MUT reporter plasmid was made by replacing the adenosine bases within the m6A consensus sequences to cytosine.

**Luciferase reporter assays**

To measure promoter activity, the cells were co-transfected with pEZX-PL01-GATA3, and KIAA1429 siRNAs or control siRNA using Lipofectamine 3000® (Invitrogen, CA, USA). For 3’ UTR luciferase reporter assays, pmirGLO-GATA3-WT or pmirGLO-GATA3-MUT was co-transfected with KIAA1429 siRNAs or control siRNA into HEK293T cells. Likewise, pmirGLO-GATA3-WT or pmirGLO-GATA3-MUT was co-transfected with GATA3-AS siRNAs or control siRNA into HEK293T cells. After 48 h of transfection, Renilla and firefly luciferase activities were detected by the Synergy Mx Multi-Mode Microplate Reader (BioTek, Vermont, USA). Firefly luciferase activity was normalized to Renilla luciferase activity and was presented as the relative luciferase activity.

**Cell transfection**

1 × 10^6^ cells per well (6-well plate) were transfected with siRNAs by using Genmute^TM^ Reagent (SignaGen Laboratories, Maryland, USA) according to the manufacturer’s protocol. GenJet^TM^ Plus reagent (SignaGen Laboratories, Maryland, USA) was used for cell transfection with plasmids. The cells were harvested at 48 h after transfection.

**Subcellular RNA fractionation**

Cytoplasmic and nuclear RNA fractions were collected according to the instructions of the PARIS^TM^ Kit (Invitrogen, CA, USA), followed by qPCR analysis. U6 small nuclear RNA was used as the nuclear endogenous control. β-actin mRNA was used as the cytoplasmic endogenous control.

**Subcellular protein fractionation**

Cytoplasmic and nuclear protein fractions were extracted according to the protocol of the NE-PER^TM^ Nuclear and Cytoplasmic Extraction Reagents (Thermo Fisher Scientific, CA, USA), after which the samples were separated on 10% SDS-PAGE gels and analyzed by Western blot. Histone H3 was used as the nuclear endogenous control. β-Tubulin was used as the cytoplasmic endogenous control.

**Western blot analysis**

Total cells and tissues lysates were prepared using RIPA Lysis Buffer (Beyotime Biotechnology, Shanghai, China) supplemented with protease inhibitor cocktail (Beyotime Biotechnology, Shanghai, China). Protein concentration was detected using the BCA Protein Assay Kit (Beyotime Biotechnology, Shanghai, China). Proteins were separated by sodium dodecyl sulfate-polyacrylamide gel electrophoresis (SDS-PAGE) and transferred to PVDF membranes (Millipore, Massachusetts, USA). After incubation with primary antibodies, the membranes were incubated with horseradish peroxidase (HRP)-conjugated secondary antibodies. Immunoreactivity was visualized using enhanced chemiluminescent (ECL) chromogenic substrate (Millipore, Massachusetts, USA). The intensity of signals was scanned by ChemiDoc MP Imager System (Bio-Rad, California, USA) and analyzed by Image Lab 5.2 software (Bio-Rad, California, USA).

**Primary antibodies used in this study:**

| Antigens | Manufacturer | Catalog Number | Application |
| --- | --- | --- | --- |
| KIAA1429  KIAA1429  KIAA1429  METTL3  METTL14  GAPDH | Novus  Proteintech  Cell Signaling Technology  Cell Signaling Technology  Cell Signaling Technology  Zen BioScience | NBP1-85118  25712-1-AP  #88358  #96391  #51104  200306 | 1:50 for IHC  1:500 for WB  1:50 for RIP  1:1000 for WB  1:1000 for WB  1:5000 for WB |
| GATA3 | Proteintech | 10417-1-AP | 1:500 for WB |
| HuR | Abcam | ab136542 | 1:1000 for WB, 1:50 for RIP |
| β-Tubulin | Affinity | AF7011 | 1:500 for WB |
| Histone H3 | Affinity | AF6359 | 1:500 for WB |
| IHC, immunohistochemistry; WB, western blot; RIP, RNA immunoprecipitation. | | | |

**Actinomycin D assays**

Cells were transfected with KIAA1429 siRNAs or Control siRNA, before being exposed to actinomycin D (1 µg/ml) for 0 h, 6 h, 12 h, 24 h and 48 h, respectively. The relative expression of GATA3 was detected by qPCR and normalized to the values measured in the 0 h group.

**m6A Quantification**

Total RNA was extracted by using Cell Total RNA Isolation Kit (Foregene, Chengdu, China) according to the manufacturer’s instructions and treated with gDNA wiper mix (Vazyme, Nanjing, China). PolyA+ mRNA was purified using Dynabeads^TM^ mRNA Purification Kit (Invitrogen, CA, USA). EpiQuik m6A RNA Methylation Quantification Kit (Colorimetric) (Epigentek, NY, USA) was utilized to measure the global m6A levels in mRNA following the manufacturer’s protocol. 200 ng polyA+ mRNA was used per each sample analysis.

**References**

1. Liu J, Lichtenberg T, Hoadley KA, Poisson LM, Lazar AJ, Cherniack AD, Kovatich AJ, Benz CC, Levine DA, Lee AV, et al: An Integrated TCGA Pan-Cancer Clinical Data Resource to Drive High-Quality Survival Outcome Analytics. *Cell* 2018, 173:400-416.e411.

2. Schefe JH, Lehmann KE, Buschmann IR, Unger T, Funke-Kaiser H: Quantitative real-time RT-PCR data analysis: current concepts and the novel "gene expression's CT difference" formula. *J Mol Med (Berl)* 2006, 84:901-910.

3. Olmeda D, Cerezo-Wallis D, Riveiro-Falkenbach E, Pennacchi PC, Contreras-Alcalde M, Ibarz N, Cifdaloz M, Catena X, Calvo TG, Canon E, et al: Whole-body imaging of lymphovascular niches identifies pre-metastatic roles of midkine. *Nature* 2017, 546:676-680.
