# Supplementary figures and images for: KIAA1429 contributes to liver cancer progression through N6-methyladenosine-dependent post-transcriptional modification of GATA3
Source: Mol Cancer. 2019 Dec 19;18:186. doi: 10.1186/s12943-019-1106-z (PMC6921542; doi:10.1186/s12943-019-1106-z)

**Figure S1**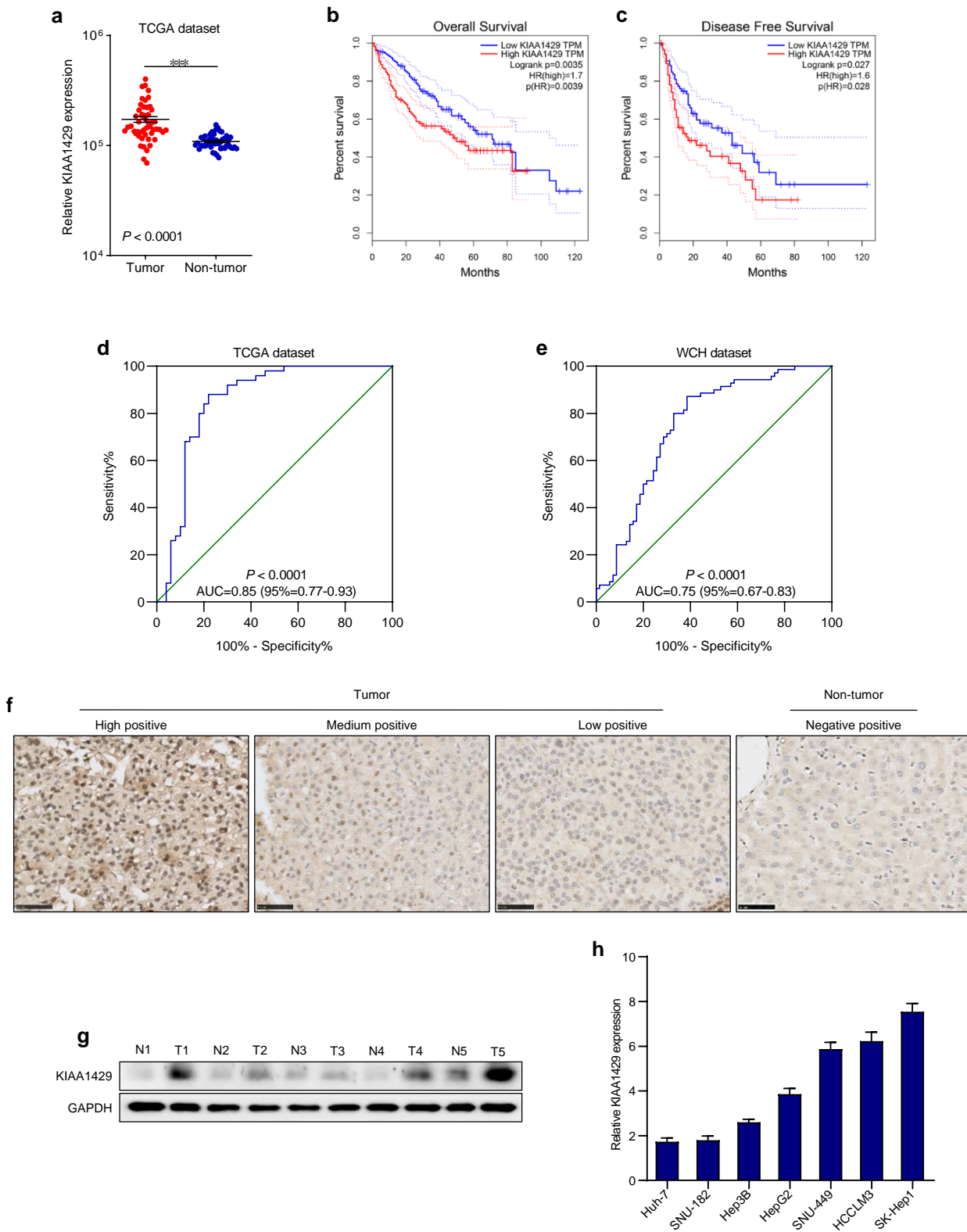

Supplement: Supplementary file 2 — Additional file 2: Figure S1. KIAA1429 is elevated in HCC tissues. a, KIAA1429 expression in 50 pairs of HCC tissues and adjacent normal tissues from TCGA dataset. b-c, Kaplan-Meier analyses of the correlations between KIAA1429 expression and overall survival or disease-free survival of 50 HCC patients. d-e, ROC analysis of KIAA1429 expression in HCC tissues and adjacent normal tissues from TCGA and WCH datasets. f, Representative IHC stains of KIAA1429 in HCC tissues and adjacent normal tissues. g, Western blot analysis of GATA3 expression in 5 pairs of HCC tissues and adjacent normal tissues. h, GATA3 expression in seven human hepatoma cell lines. Data are presented as mean ± SEM. [file 12943_2019_1106_MOESM2_ESM.pdf]

**Figure S2**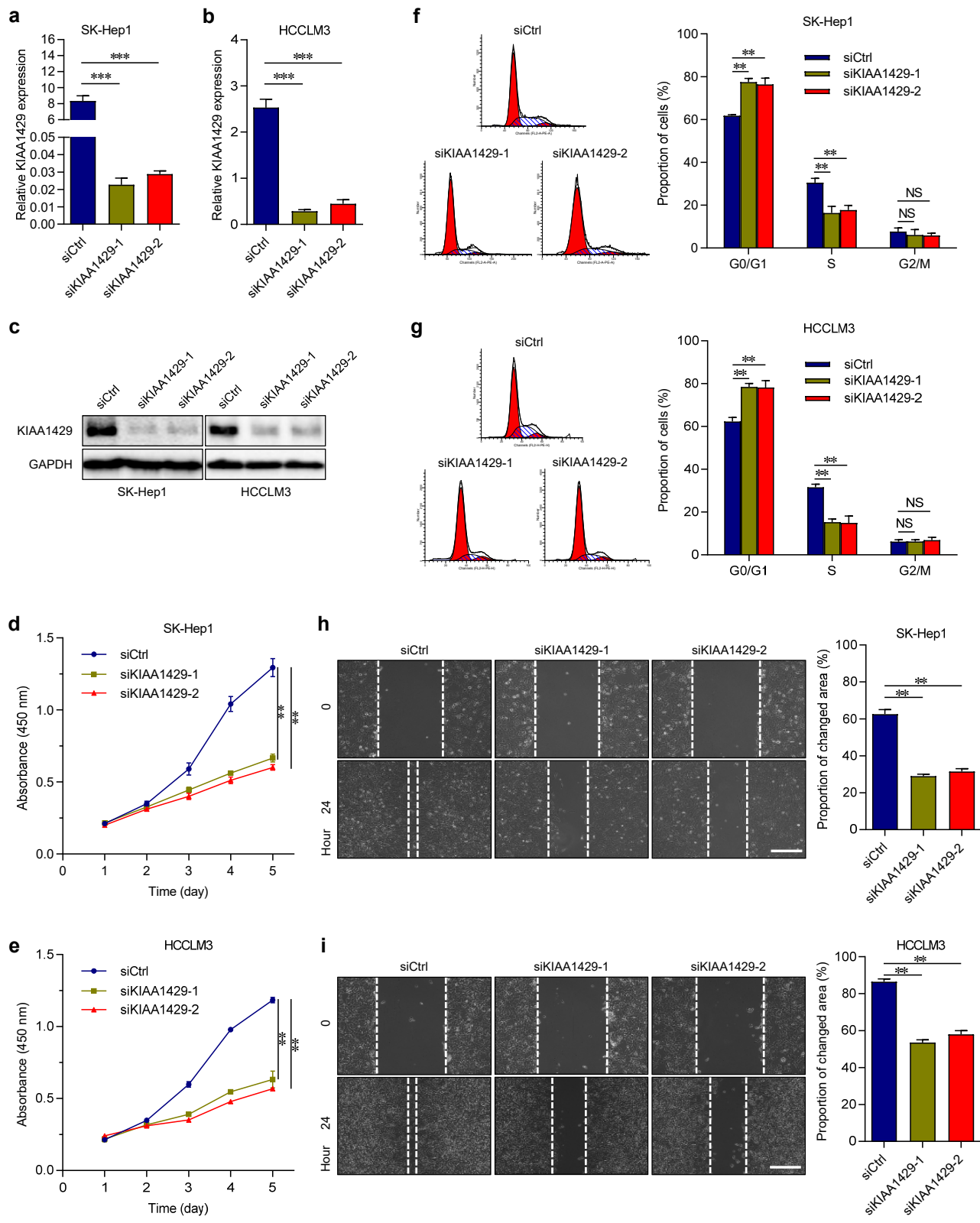

Supplement: Supplementary file 4 — Additional file 4: Figure S2. Inhibition of KIAA1429 impairs the cell proliferation and migration in vitro. a-b, GATA3 expression in SK-Hep1 and HCCLM3 cells transfected with KIAA1429 siRNAs or the control by using qPCR. c, Western blot analysis of GATA3 expression in SK-Hep1 and HCCLM3 cells transfected with KIAA1429 siRNAs or the control. d-e, CCK-8 assays for SK-Hep1 and HCCLM3 cells transfected with KIAA1429 siRNAs or the control. f-g, Cell cycle distribution was measured by PI staining in SK-Hep1 and HCCLM3 cells transfected with KIAA1429 siRNAs or the control, followed by flow cytometric analysis. h-i, Wound-healing migration assays for SK-Hep1 and HCCLM3 cells transfected with KIAA1429 siRNAs or the control. Scale bars = 100 μm. Data are presented as mean ± SEM. **P < 0.01. [file 12943_2019_1106_MOESM4_ESM.pdf]

**Figure S3**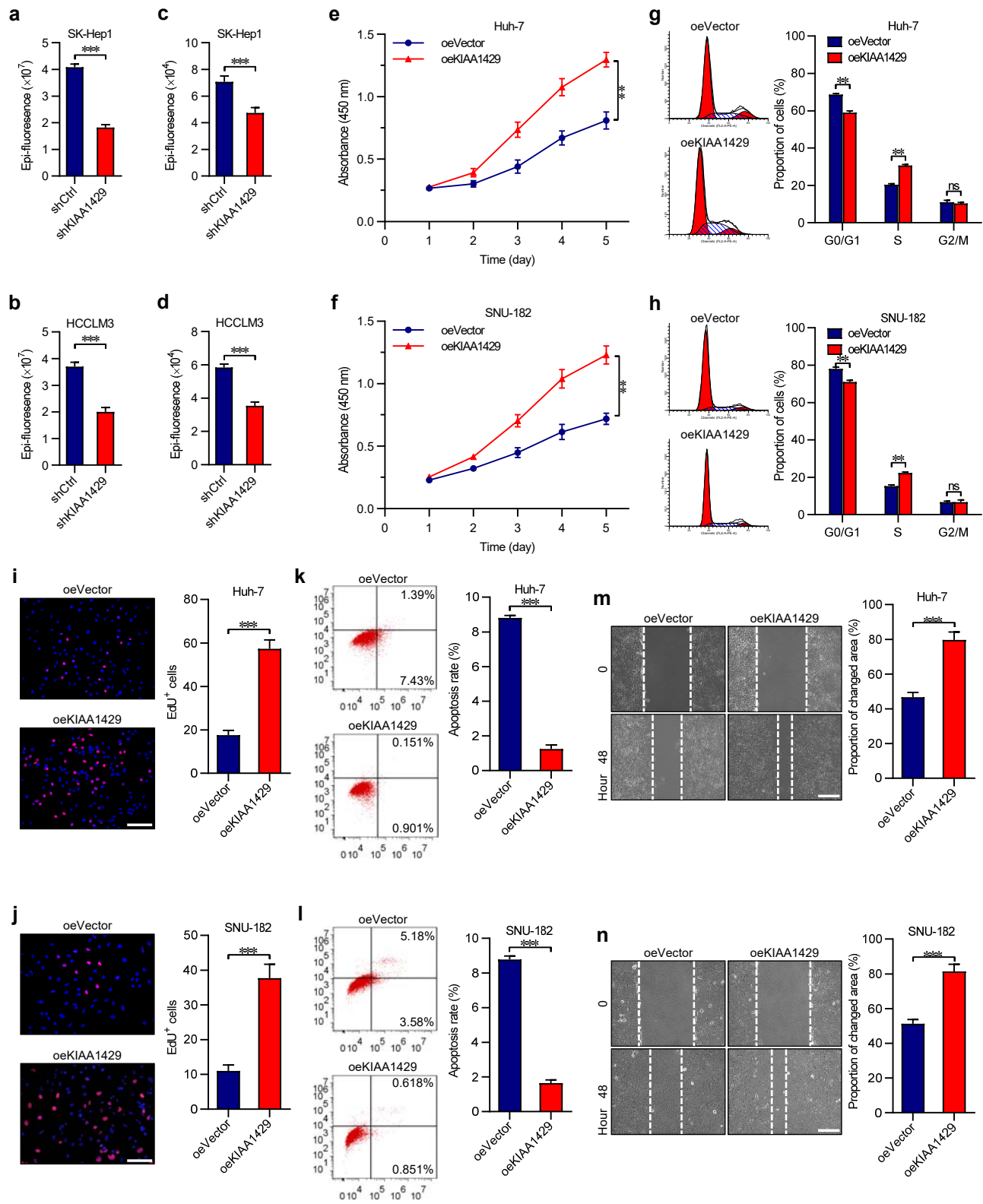

Supplement: Supplementary file 5 — Additional file 5: Figure S3. Overexpression of KIAA1429 promotes the cell proliferation and metastasis in vitro. a-b, Fluoresence signal intensities of livers in each group after orthotopic implantation with indicated SK-Hep1 and HCCLM3 cells. c-d, Fluoresence signal intensities of lungs in each group after tail intravenous injection with indicated SK-Hep1 and HCCLM3 cells. e-f, CCK-8 assays for Huh-7 and SNU-182 cells with or without KIAA1429 upregulation. g-h, Cell cycle distribution was measured by PI staining in Huh-7 and SNU-182 cells with or without KIAA1429 upregulation, followed by flow cytometric analysis. i-j, EdU immunofluorescence staining assays for Huh-7 and SNU-182 cells with or without KIAA1429 upregulation. Scale bars = 100 μm. k-l, Cell apoptosis was measured by FITC-Annexin V and PI staining in Huh-7 and SNU-182 cells with or without KIAA1429 upregulation, followed by flow cytometric analysis. m-n, Wound-healing migration assays for Huh-7 and SNU-182 cells with or without KIAA1429 upregulation. Scale bars = 100 μm. Data are presented as mean ± SEM. NS: not significant; **P < 0.01, ***P < 0.001. [file 12943_2019_1106_MOESM5_ESM.pdf]

Figure S4

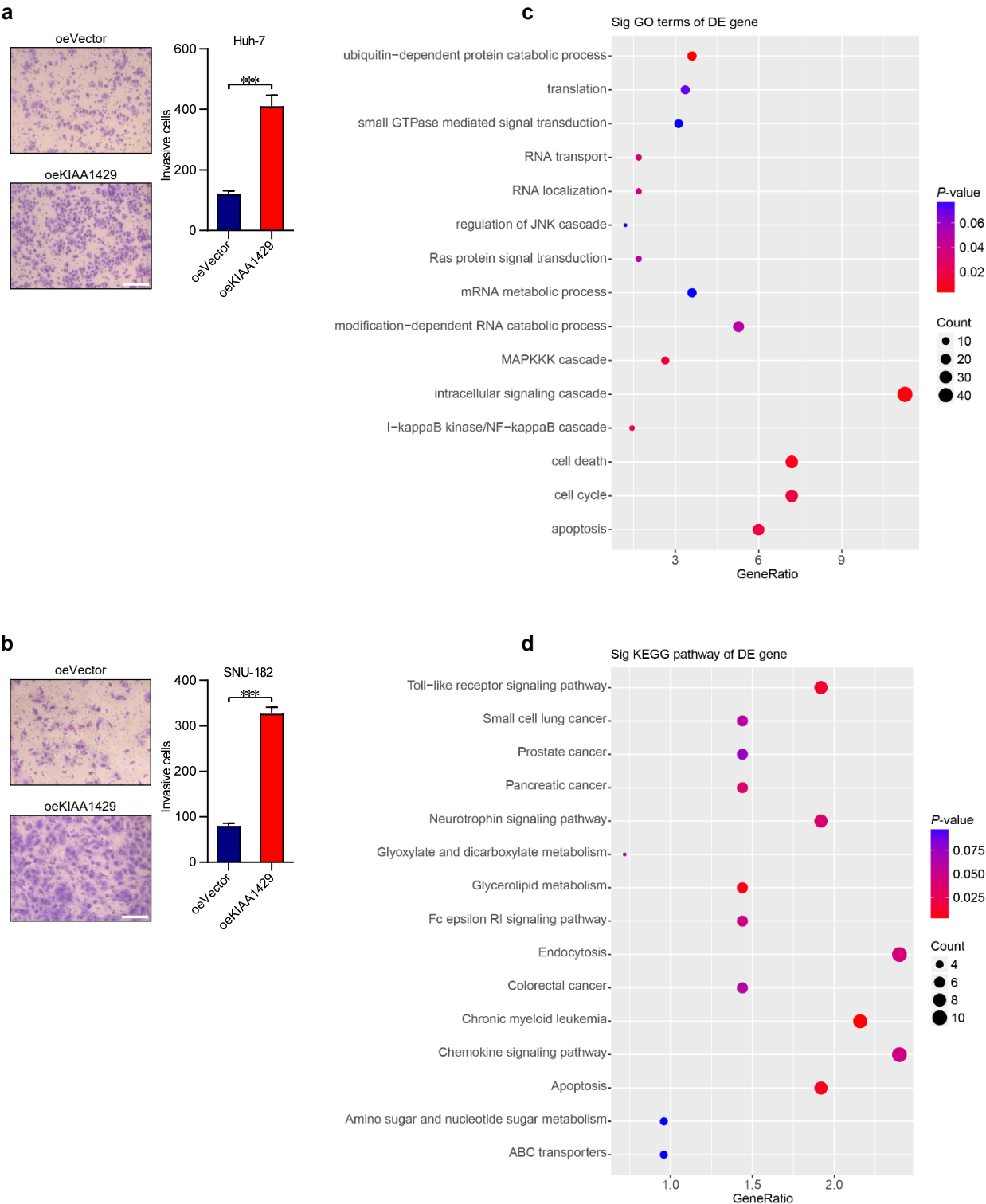

Supplement: Supplementary file 6 — Additional file 6: Figure S4. Analysis of differentially expressed genes by KIAA1429 knockdown in RNA-seq. a-b, Transwell invasion assays for Huh-7 and SNU-182 cells with or without KIAA1429 upregulation. Scale bars = 100 μm. c-d, Gene Ontology and KEGG pathway analysis of the differentially expressed genes by KIAA1429 knockdown in RNA-seq. Data are presented as mean ± SEM. ***P < 0.001. [file 12943_2019_1106_MOESM6_ESM.pdf]

**Figure S5**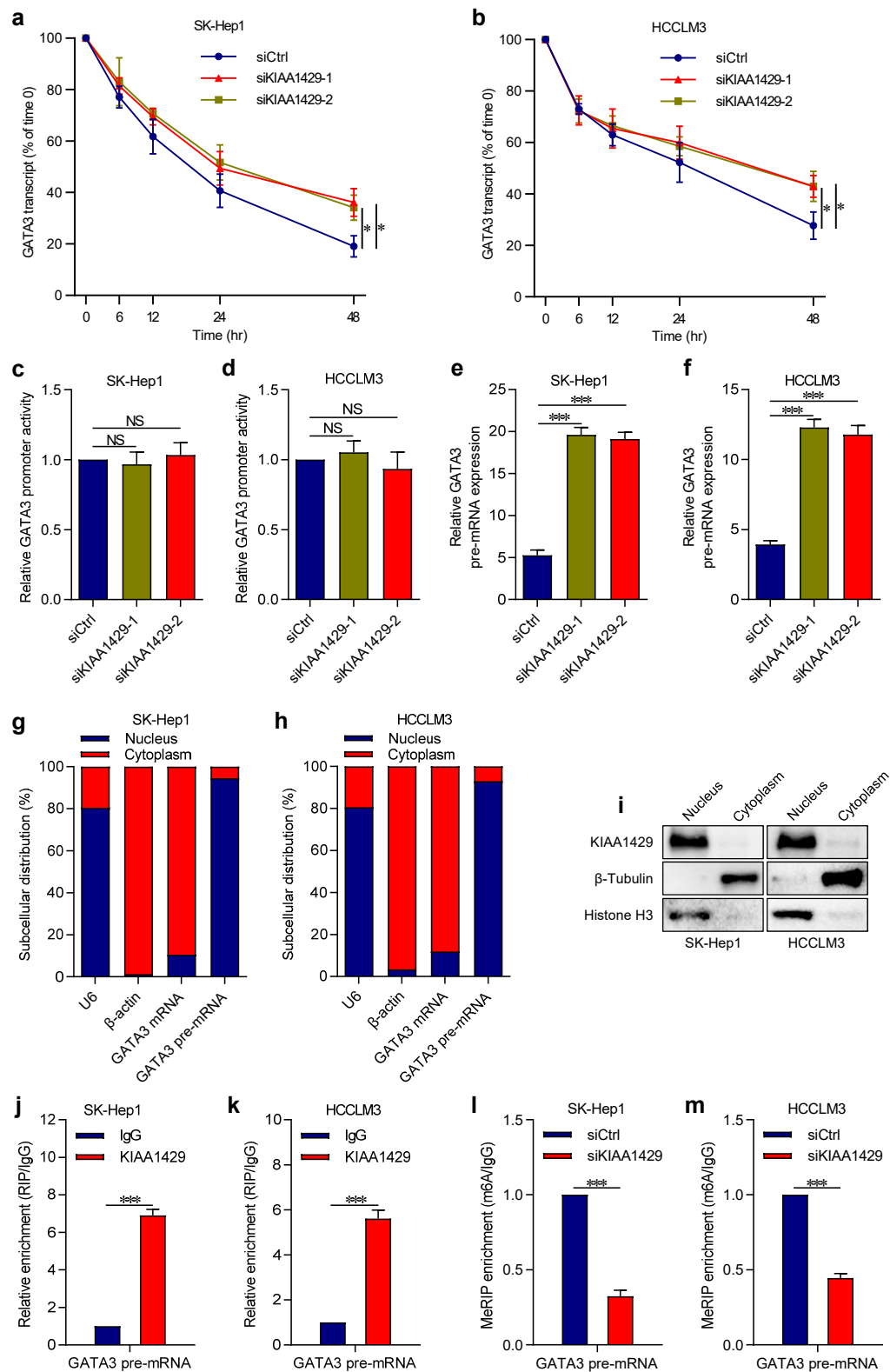

Supplement: Supplementary file 7 — Additional file 7: Figure S5. KIAA1429 mediates the m6A modification of GATA3 pre-mRNA. a-b, The RNA levels of GATA3 mRNA at the indicated time points were analyzed by qPCR relative to time 0 after blocking new RNA synthesis with actinomycin D (1 mg/mL) in SK-Hep1 and HCCLM3 cells and normalized to 18S rRNA. c-d, Relative luciferase activity of the GATA3 promoter firefly luciferase reporter in SK-Hep1 and HCCLM3 cells transfected with KIAA1429 siRNAs or the control. Data are shown as the relative ratio of firefly luciferase activity to renilla luciferase activity. e-f, GATA3 pre-mRNA expression in SK-Hep1 and HCCLM3 cells transfected with KIAA1429 siRNAs or the control. g-h, RNA distribution analysis of GATA3 mRNA and GATA3 pre-mRNA in subcellular fractions of SK-Hep1 and HCCLM3 cells assessed by qPCR. U6 served as the nuclear marker, β-actin served as cytoplasmic marker. i, Protein distribution analysis of KIAA1429 in subcellular fractions of SK-Hep1 and HCCLM3 cells assessed by western blot. Histone H3 served as the nuclear marker, β-Tubulin served as cytoplasmic marker. j-k, RIP-seq of the enrichment of GATA3 pre-mRNA on KIAA1429 relative to IgG. l-m, MeRIP-qPCR analysis of GATA3 pre-mRNA in SK-Hep1 and HCCLM3 cells transfected with KIAA1429 siRNAs or the control. Data are presented as mean ± SEM. NS: not significant; *P < 0.05, ***P< 0.001. [file 12943_2019_1106_MOESM7_ESM.pdf]

**Figure S6**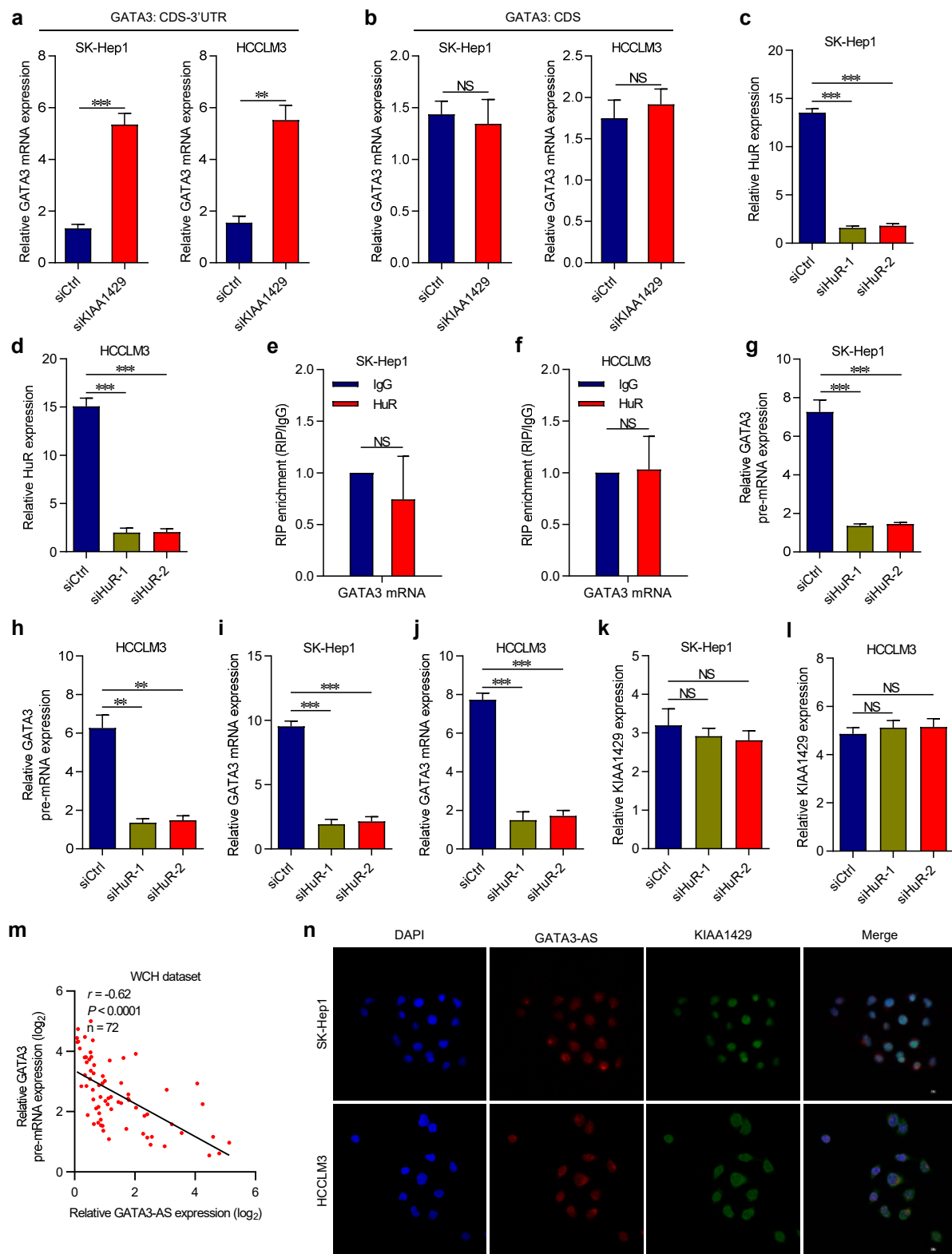

Supplement: Supplementary file 8 — Additional file 8: Figure S6. HuR mediates the regulation of GATA3 by KIAA1429. a-b, GATA3 expression in SK-Hep1 and HCCLM3 cells co-transfected with GATA3 CDS-3’ UTR or GATA3 CDS vector and KIAA1429 siRNAs or the control by using qPCR. c-d, HuR expression in SK-Hep1 and HCCLM3 cells transfected with HuR siRNAs or the control by using qPCR. e-f, RIP-seq of the enrichment of GATA3 mRNA on HuR relative to IgG. g-h, GATA3 pre-mRNA expression in SK-Hep1 and HCCLM3 cells transfected with HuR siRNAs or the control by using qPCR. i-j, GATA3 mRNA expression in SK-Hep1 and HCCLM3 cells transfected with HuR siRNAs or the control by using qPCR. k-l, KIAA1429 expression in SK-Hep1 and HCCLM3 cells transfected with HuR siRNAs or the control by using qPCR. m, Scatter plots of GATA3-AS versus GATA3 pre-mRNA expression in WCH data repository. Pearson correlation coefficients (r) and P values are shown. n, Combined application of FISH and IF. LNA probes for GATA3-AS (red) and a fluorescence-conjugatedsecondary antibody was used for KIAA1429 (green). Data are presented as mean ± SEM. NS: not significant; **P < 0.01, ***P < 0.001. [file 12943_2019_1106_MOESM8_ESM.pdf]

**Figure S7**

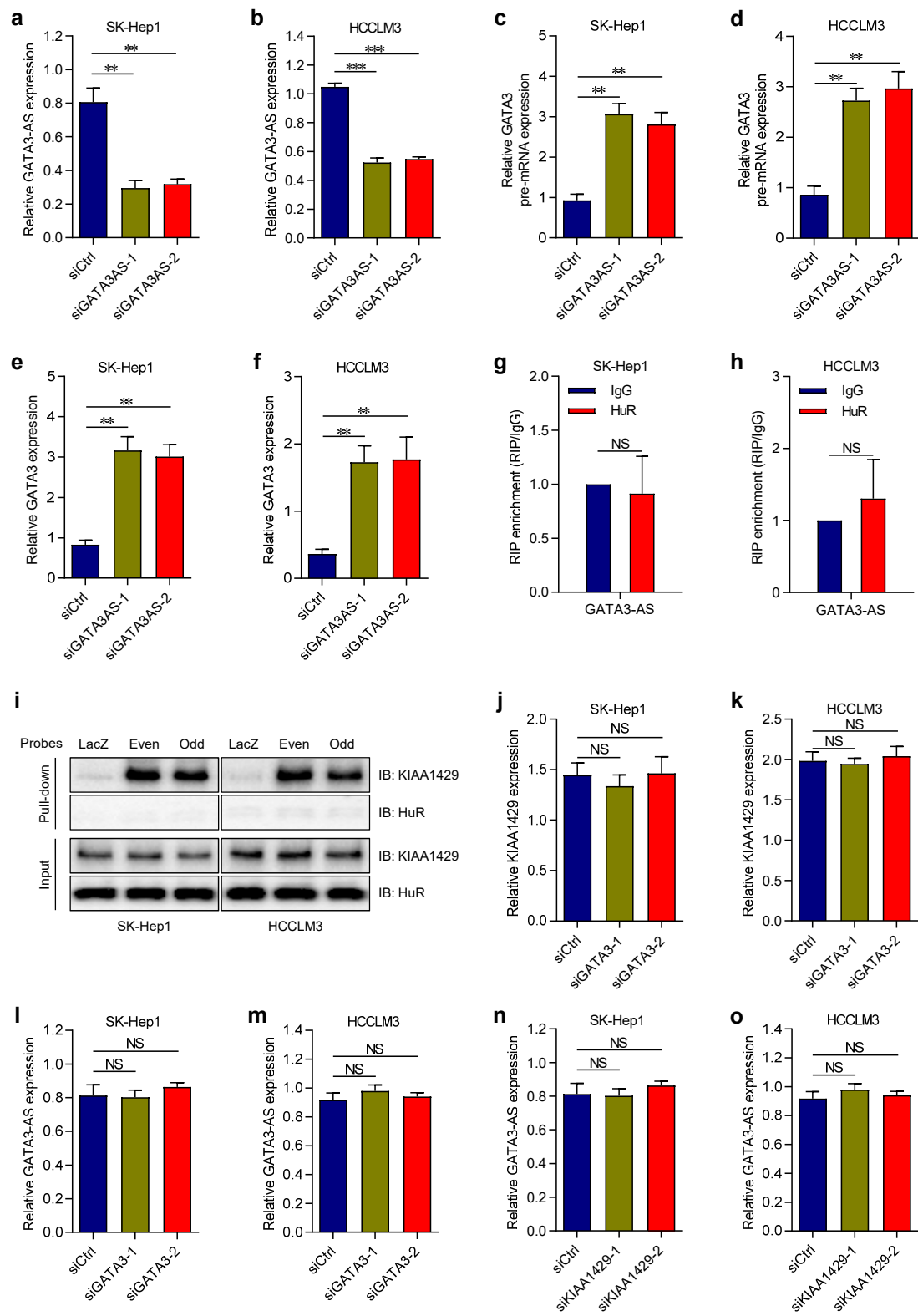

Supplement: Supplementary file 9 — Additional file 9: Figure S7. GATA3-AS functions as a guide lncRNA that targetedly promotes the interaction of KIAA1429 with GATA3 pre-mRNA. a-b, GATA3-AS expression in SK-Hep1 and HCCLM3 cells transfected with GATA3-AS siRNAs or the control by using qPCR. c-d, GATA3 pre-mRNA expression in SK-Hep1 and HCCLM3 cells transfected with GATA3-AS siRNAs or the control by using qPCR. e-f, GATA3 mRNA expression in SK-Hep1 and HCCLM3 cells transfected with GATA3-AS siRNAs or the control by using qPCR. g-h, RIP-seq of the enrichment of GATA3-AS on HuR relative to IgG. i, Western blot analysis of HuR in protein samples pulled down by even and odd sets for GATA3-AS, and control LacZ probes pool in SK-Hep1 and HCCLM3 cells. j-k, KIAA1429 expression in SK-Hep1 and HCCLM3 cells transfected with GATA3 siRNAs or the control by using qPCR. l-m, GATA3-AS expression in SK-Hep1 and HCCLM3 cells transfected with GATA3 siRNAs or the control by using qPCR. n-o, GATA3-AS expression in SK-Hep1 and HCCLM3 cells transfected with KIAA1429 siRNAs or the control by using qPCR. Data are presented as mean ± SEM. NS: not significant; **P < 0.01, ***P < 0.001. [file 12943_2019_1106_MOESM9_ESM.pdf]

**Figure S8**

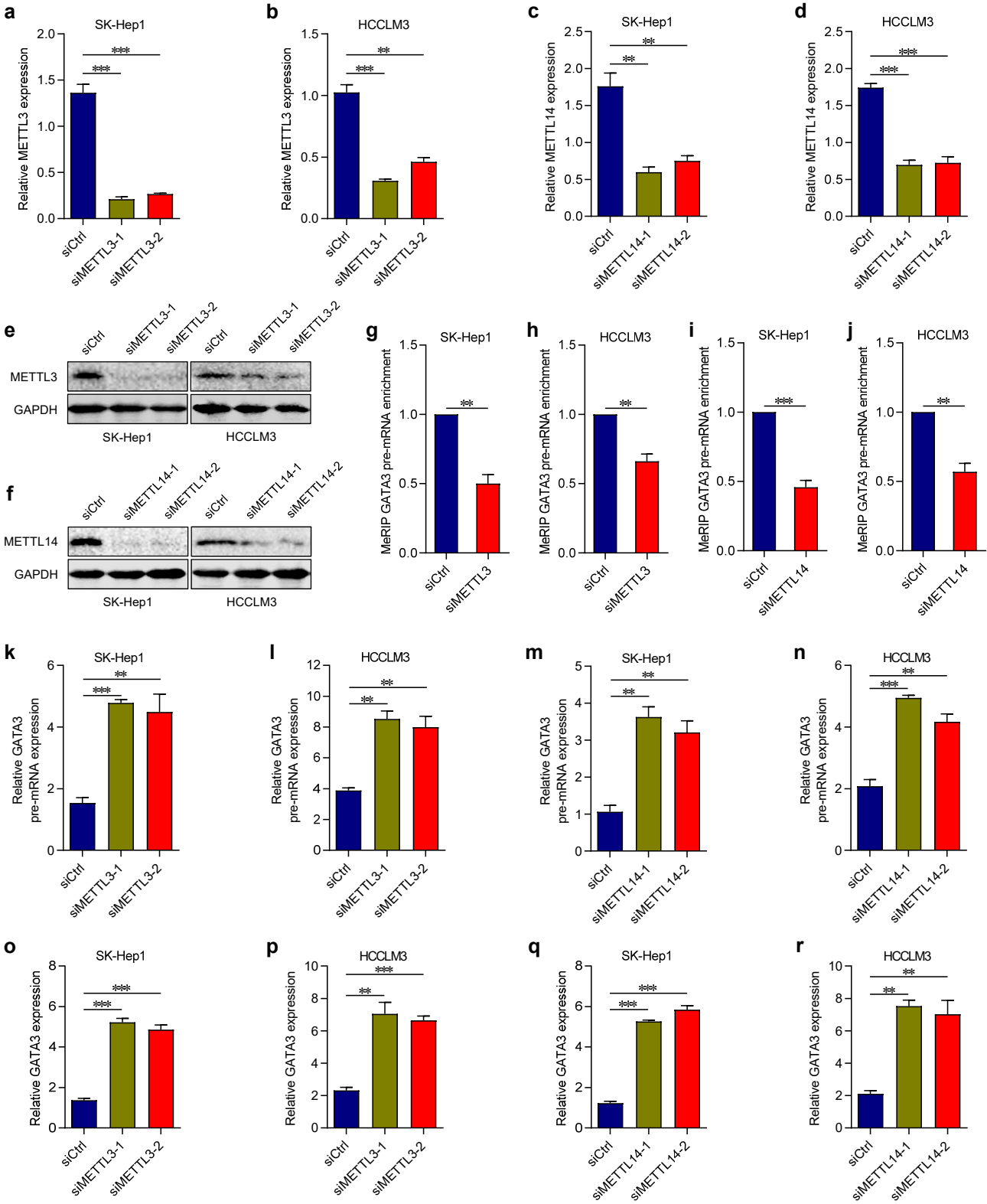

Supplement: Supplementary file 10 — Additional file 10: Figure S8. Knockdown of METTL3 or METTL14 regulates the m6a modification and expression levels of GATA3. a-d, METTL3 or METTL14 expression in SK-Hep1 and HCCLM3 cells transfected with METTL3 or METTL14 siRNAs or the control by using qPCR. e-f, Western blot analysis of METTL3 or METTL14 expression in SK-Hep1 and HCCLM3 cells with METTL3 or METTL14 siRNAs or the control. g-j, MeRIP-qPCR analysis of GATA3 pre-mRNA in SK-Hep1 and HCCLM3 cells transfected with METTL3 or METTL14 siRNAs or the control. k-r, GATA3 pre-mRNA or mRNA expression in SK-Hep1 and HCCLM3 cells transfected with METTL3 or METTL14 siRNAs or the control by using qPCR. Data are presented as mean ± SEM. **P < 0.01, ***P < 0.001. [file 12943_2019_1106_MOESM10_ESM.pdf]

**Figure S9**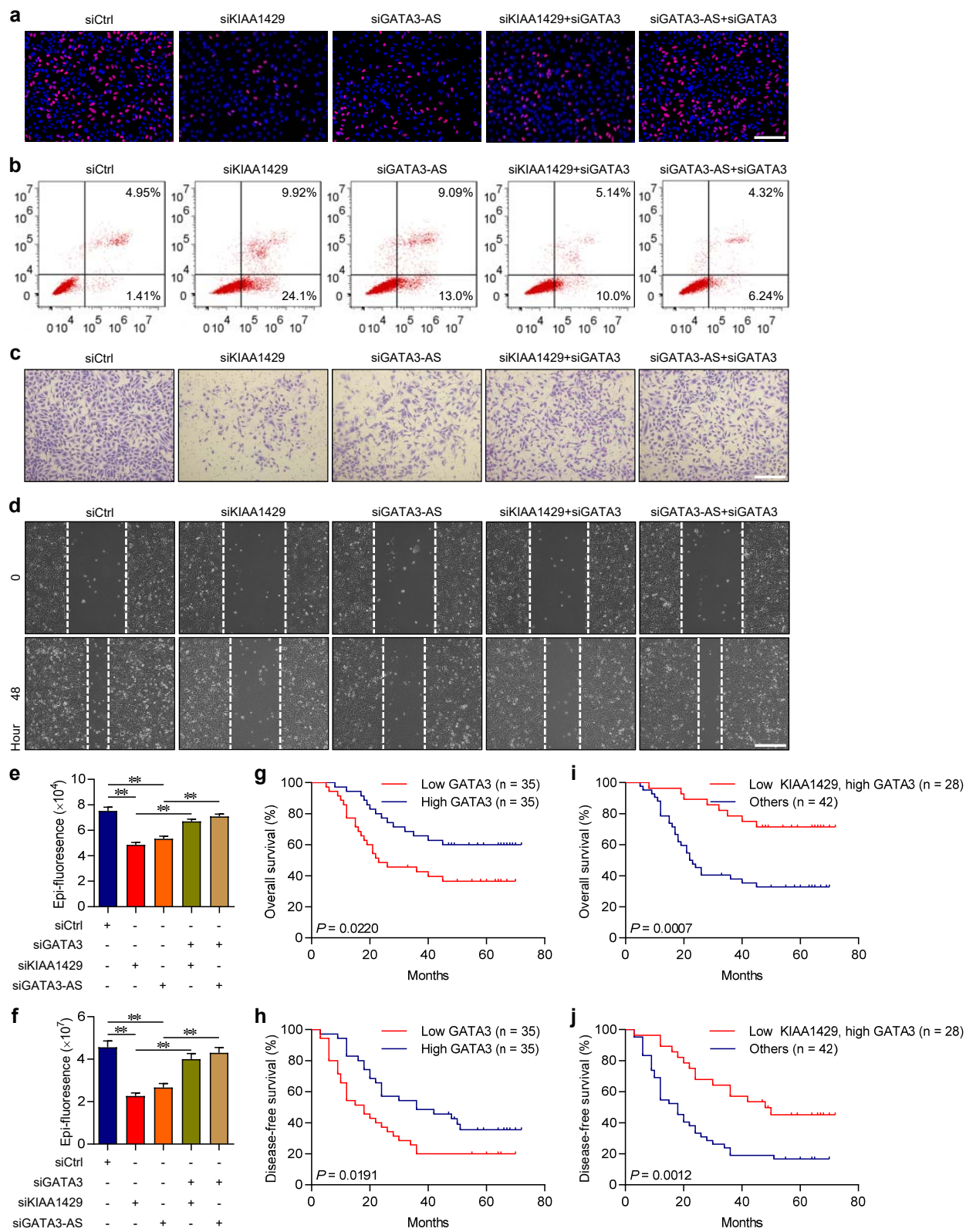

Supplement: Supplementary file 11 — Additional file 11: Figure S9. GATA3 mediates the cell proliferation and metastasis driven by KIAA1429 or GATA3-AS in vitro. a, EdU immunofluorescence staining assays for indicated cells. Scale bars = 100 μm. b, Cell apoptosis was measured by FITC-Annexin V and PI staining in indicated cells, followed by flow cytometric analysis. c, Transwell invasion assays for indicated cells. Scale bars = 100 μm. d, Wound-healing migration assays for indicated cells. Scale bars = 100 μm. e, Fluoresence signal intensities of livers in each group after orthotopic implantation with indicated cells. f, Fluoresence signal intensities of lungs in each group after tail intravenous injection with indicated cells. g-h, Kaplan-Meier analyses of the correlations between GATA3 expression and overall survival or disease-free survival of 70 HCC patients. The median expression level was used as the cutoff. Values are expressed as the median with interquartile range. i-j, Kaplan-Meier analyses of the overall survival or disease-free survival between patients with low KIAA1429 and high GATA3 expressions and others. Data are presented as mean ± SEM. **P < 0.01. [file 12943_2019_1106_MOESM11_ESM.pdf]
