# Supplementary material for: KIAA1429 contributes to liver cancer progression through N6-methyladenosine-dependent post-transcriptional modification of GATA3
Source: Mol Cancer. 2019 Dec 19;18:186. doi: 10.1186/s12943-019-1106-z (PMC6921542; doi:10.1186/s12943-019-1106-z)
Supplement: Supplementary file 3 — Additional file 3: Table S1. Clinical characteristics of 70 HCC patients according to KIAA1429 expression level. [file 12943_2019_1106_MOESM3_ESM.docx]

| **Table S1.** Clinical characteristics of 70 HCC patients according to KIAA1429 expression level | | | | |
| --- | --- | --- | --- | --- |
| Variable | Total no. (n=70) | KIAA1429 | | *P*-value |
|  |  | High (n=35) | Low (n=35) |  |
| Gender | | | | |
| Male | 61 | 33 | 28 | 0.1532 |
| Female | 9 | 2 | 7 |  |
| Age, years | |  |  |  |
| >50 | 39 | 17 | 22 | 0.3358 |
| ≤50 | 31 | 18 | 13 |  |
| Tumor size, cm | | | | |
| >5 | 39 | 24 | 15 | 0.0303* |
| ≤5 | 31 | 11 | 20 |  |
| No. tumor | | | | |
| multiple | 20 | 11 | 9 | 0.5967 |
| solitary | 50 | 24 | 26 |  |
| Serum AFP, µg/L | | | | |
| >20 | 40 | 25 | 15 | 0.0157* |
| ≤20 | 30 | 10 | 20 |  |
| Liver cirrhosis | | | | |
| Present | 33 | 19 | 14 | 0.2312 |
| Absent | 37 | 16 | 21 |  |
| Microvascular invasion | | | | |
| Present | 34 | 22 | 12 | 0.0168* |
| Absent | 36 | 13 | 23 |  |
| Edmondson’s grade | | | | |
| III+IV | 34 | 15 | 19 | 0.3388 |
| I+II | 36 | 20 | 16 |  |
| TNM stage | | | | |
| II+III | 47 | 28 | 19 | 0.0220* |
| I | 23 | 7 | 16 |  |
| BCLC stage | | | | |
| B+C | 47 | 29 | 18 | 0.0051* |
| A | 23 | 6 | 17 |  |
|  | | | | |
