# Supplementary material for: KIAA1429 contributes to liver cancer progression through N6-methyladenosine-dependent post-transcriptional modification of GATA3
Source: Mol Cancer. 2019 Dec 19;18:186. doi: 10.1186/s12943-019-1106-z (PMC6921542; doi:10.1186/s12943-019-1106-z)
Supplement: Supplementary file 13 — Additional file 13: Table S3. Univariate analysis of several variables for OS. [file 12943_2019_1106_MOESM13_ESM.docx]

| **Table S3.** Univariate analysis of several variables for OS | | |
| --- | --- | --- |
| Variable | Hazard ratio (95%CI) | *P*-value |
| Gender | - | 0.236 |
| Age, years | - | 0.091 |
| Tumor size, cm | 1.247 (1.150-1.352) | <0.001* |
| No. tumor | - | 0.070 |
| Serum AFP, µg/L | - | 0.889 |
| Liver cirrhosis | - | 0.795 |
| Microvascular invasion | 2.929 (1.478-5.806) | 0.002* |
| Edmondson’s grade | - | 0.290 |
| TNM stage | 6.254 (2.198-17.794) | 0.001* |
| BCLC stage | 8.923 (2.721-29.264) | <0.001* |
| KIAA1429 | 3.457 (2.052-5.823) | <0.001* |
| GATA3 | 0.825 (0.737-0.924) | 0.001* |
|  |  |  |
