# Supplementary material for: KIAA1429 contributes to liver cancer progression through N6-methyladenosine-dependent post-transcriptional modification of GATA3
Source: Mol Cancer. 2019 Dec 19;18:186. doi: 10.1186/s12943-019-1106-z (PMC6921542; doi:10.1186/s12943-019-1106-z)
Supplement: Supplementary file 14 — Additional file 14: Table S4. Multivariate analysis of several variables for OS. [file 12943_2019_1106_MOESM14_ESM.docx]

| **Table S4.** Multivariate analysis of several variables for OS | | |
| --- | --- | --- |
| Variable | Hazard ratio (95%CI) | *P*-value |
| Gender | - | 0.236 |
| Age, years | - | 0.091 |
| Tumor size, cm | 1.237 (1.126-1.360) | <0.001* |
| No. tumor | - | 0.070 |
| Serum AFP, µg/L | - | 0.889 |
| Liver cirrhosis | - | 0.795 |
| Microvascular invasion | - | 0.084 |
| Edmondson’s grade | - | 0.290 |
| TNM stage | - | 0.054 |
| BCLC stage | 4.818 (1.414-16.421) | 0.012* |
| KIAA1429 | - | 0.306 |
| GATA3 | 0.851 (0.756-0.956) | 0.007* |
|  |  |  |
