# Supplementary material for: KIAA1429 contributes to liver cancer progression through N6-methyladenosine-dependent post-transcriptional modification of GATA3
Source: Mol Cancer. 2019 Dec 19;18:186. doi: 10.1186/s12943-019-1106-z (PMC6921542; doi:10.1186/s12943-019-1106-z)
Supplement: Supplementary file 15 — Additional file 15: Table S5. Univariate analysis of several variables for DFS. [file 12943_2019_1106_MOESM15_ESM.docx]

| **Table S5.** Univariate analysis of several variables for DFS | | |
| --- | --- | --- |
| Variable | Hazard ratio (95%CI) | *P*-value |
| Gender | - | 0.282 |
| Age, years | - | 0.131 |
| Tumor size, cm | 1.201 (1.116-1.294) | <0.001* |
| No. tumor | 2.197 (1.234-3.912) | 0.007* |
| Serum AFP, µg/L | - | 0.858 |
| Liver cirrhosis | - | 0.675 |
| Microvascular invasion | 2.399 (1.363-4.222) | 0.002* |
| Edmondson’s grade | - | 0.395 |
| TNM stage | 4.136 (1.988-8.604) | <0.001* |
| BCLC stage | 5.244 (2.426-11.333) | <0.001* |
| KIAA1429 | 3.063 (1.903-4.931) | <0.001* |
| GATA3 | 0.889 (0.833-0.948) | <0.001* |
|  |  |  |
