# Supplementary material for: KIAA1429 contributes to liver cancer progression through N6-methyladenosine-dependent post-transcriptional modification of GATA3
Source: Mol Cancer. 2019 Dec 19;18:186. doi: 10.1186/s12943-019-1106-z (PMC6921542; doi:10.1186/s12943-019-1106-z)
Supplement: Supplementary file 16 — Additional file 16: Table S6. Multivariate analysis of several variables for DFS. [file 12943_2019_1106_MOESM16_ESM.docx]

| **Table S6.** Multivariate analysis of several variables for DFS | | |
| --- | --- | --- |
| Variable | Hazard ratio (95%CI) | *P*-value |
| Gender | - | 0.282 |
| Age, years | - | 0.131 |
| Tumor size, cm | 1.161 (1.068-1.262) | <0.001* |
| No. tumor | - | 0.392 |
| Serum AFP, µg/L | - | 0.858 |
| Liver cirrhosis | - | 0.675 |
| Microvascular invasion | - | 0.213 |
| Edmondson’s grade | - | 0.395 |
| TNM stage | 2.281 (1.037-5.017) | 0.040* |
| BCLC stage | 2.775 (1.193-6.455) | 0.018* |
| KIAA1429 | - | 0.617 |
| GATA3 | 0.906 (0.848-0.967) | 0.003* |
|  |  |  |
