# Supplementary material for: KIAA1429 contributes to liver cancer progression through N6-methyladenosine-dependent post-transcriptional modification of GATA3
Source: Mol Cancer. 2019 Dec 19;18:186. doi: 10.1186/s12943-019-1106-z (PMC6921542; doi:10.1186/s12943-019-1106-z)
Supplement: Supplementary file 17 — Additional file 17. Additional file legends. [file 12943_2019_1106_MOESM17_ESM.pdf]

Editing Certificate

This document certifies that the manuscript

KIAA1429 contributes to liver cancer progression through N6-methyladenosine-dependent post-transcriptional modification of GATA3

prepared by the authors

Tian Lan, Hui Li, Delin Zhang, Lin Xu, Hailing Liu, Xiangyong Hao, Xiaokai Yan, Haotian Liao, Xiangzheng Chen, Kunlin Xie, Jiaxin Li, Mingheng Liao,...

was edited for proper English language, grammar, punctuation, spelling, and overall style by one or more of the highly qualified native English speaking editors at AJE.

This certificate was issued on **November 6, 2019** and may be verifed on the AJE website using the verification code **87F7-2CC0-FA3C-6722-7FE4**.

Neither the research content nor the authors' intentions were altered in any way during the editing process. Documents receiving this certification should be English-ready for publication; however, the author has the ability to accept or reject our suggestions and changes. To verify the final AJE edited version, please visit our verification page at aje.com/certificate. If you have any questions or concerns about this edited document, please contact AJE at support@aje.com.

AJE provides a range of editing, translation, and manuscript services for researchers and publishers around the world. For more information about our company, services, and partner discounts, please visit aje.com.
